# Supplementary material for: Women’s experiences of emergency post-abortion care at Kawempe National Referral Hospital, Uganda: A qualitative descriptive study
Source: PLoS One. 2026 Nov 5;21(11):e0357615. doi: 10.1371/journal.pone.0357615 (PMC13739178; doi:10.1371/journal.pone.0357615)
Supplement: Supplementary file 2 [file pone.0357615.s003.pdf]

PLOS One

Women's experiences of emergency post-abortion care at Kawempe National Referral Hospital, Uganda: a qualitative phenomenological study

--Manuscript Draft--

**Manuscript Number:**	PONE-D-26-31923
**Article Type:**	Research Article
**Full Title:**	Women's experiences of emergency post-abortion care at Kawempe National Referral Hospital, Uganda: a qualitative phenomenological study
**Short Title:**	Women's experiences of emergency post-abortion care at Kawempe National Referral Hospital.
**Corresponding Author:**	Kawungu Saad Sessimba, MBChB, MMed, MSc PH, PgDip SRH, MSc SRH<br>Makerere University Faculty of Medicine: Makerere University College of Health Sciences<br>kampala, UGANDA
**Keywords:**	Keywords: Emergency post-abortion care; qualitative research; phenomenology; women's experience; reproductive health; Uganda
**Abstract:**	Background: Post-abortion care (PAC) encompasses emergency treatment, counselling, contraceptive services, and referral linkages. Emergency post-abortion care (EPAC), the life-saving component of PAC, addresses acute abortion-related complications, including haemorrhage, sepsis, retained products of conception, and severe pain. In Uganda, where abortion is legally restricted and socially stigmatised, women's care experiences are shaped by clinical urgency, fear, moral vulnerability, provider interactions, and structural health system constraints. Despite EPAC's centrality to maternal survival, qualitative evidence on how women interpret and evaluate their care experiences in referral hospital settings in Uganda remains limited. This study explored women's experiences of EPAC at Kawempe National Referral Hospital (KNRH) and identified the factors that shaped those experiences.<br>Methods: A qualitative phenomenological design was employed. Sixteen in-depth interview transcripts from women who received EPAC at KNRH in March–April 2026 were analysed using inductive thematic analysis. The Socio-Ecological Model (SEM) was applied as an interpretive framework.<br>Results: Six themes were identified: (1) survival and physical relief as the immediate measure of good care; (2) pain, fear, and emotional distress during treatment; (3) reassurance and support as buffers against vulnerability; (4) dignity under pressure: communication and privacy in EPAC; (5) structural barriers across the pathway of care; and (6) experiences beyond discharge: incomplete recovery and uncertainty. Care was frequently evaluated through the lens of survival, yet these accounts co-existed with intense procedural pain, compromised privacy, delays, financial burden, and inadequate post-discharge support. EPAC at KNRH was experienced as a complex encounter shaped by bodily vulnerability, interpersonal dynamics, and system-level constraints.<br>Conclusions: Strengthening EPAC requires patient-centred approaches that integrate clinical effectiveness with respectful communication, pain management, improved triage, and structured post-discharge support.
**Order of Authors:**	Kawungu Saad Sessimba, MBChB, MMed, MSc PH, PgDip SRH, MSc SRH
	Aburi Godfrey James, MBChB, MMED
	Baluku Andrew, MBChB, MMED
	Igirimbabazi Pious, MBChB, MPH, MMED
	Kibuuka Balikudembe, MBChB, MMED
	Keesiga Annette, MBChB, MMED, MPH
	Herbert Kayiga, MBChB, MMED, MPH, Fellowship, PhD
**Additional Information:**	

Powered by Editorial Manager® and ProduXion Manager® from Aries Systems Corporation

Question	Response
**Financial Disclosure**<br><br>Enter a financial disclosure statement that describes the sources of funding for the work included in this submission. Review the submission guidelines for detailed requirements. View published research articles from *PLOS ONE* for specific examples.<br><br>This statement is required for submission and **will appear in the published article** if the submission is accepted. Please make sure it is accurate.<br><br>**Funded studies**<br>Enter a statement with the following details:<br>• Initials of the authors who received each award<br>• Grant numbers awarded to each author<br>• The full name of each funder<br>• URL of each funder website<br>• Did the sponsors or funders play any role in the study design, data collection and analysis, decision to publish, or preparation of the manuscript?<br><br>Did you receive funding for this work?	The author(s) received no specific funding for this work.
**Competing Interests**<br><br>Use the instructions below to enter a competing interest statement for this submission. On behalf of all authors, disclose any competing interests that could be perceived to bias this work—acknowledging all financial support and any other relevant financial or non-financial competing interests.<br><br>This statement is required for submission and **will appear in the published article** if the submission is accepted. Please make sure it is accurate and that any funding sources listed in your Funding Information later in the submission form are also	The authors have declared that no competing interests exist.

declared in your Financial Disclosure statement.<br><br>View published research articles from *PLOS ONE* for specific examples.<br><br>**NO authors have competing interests**<br><br>Enter: *The authors have declared that no competing interests exist.*<br><br>**Authors with competing interests**<br><br>Enter competing interest details beginning with this statement:<br><br>*I have read the journal's policy and the authors of this manuscript have the following competing interests: [insert competing interests here]*<br><br>* typeset	
**Ethics Statement**<br><br>Enter an ethics statement for this submission. This statement is required if the study involved:<br><br>• Human participants<br>• Human specimens or tissue<br>• Vertebrate animals or cephalopods<br>• Vertebrate embryos or tissues<br>• Field research<br><br>Write "N/A" if the submission does not require an ethics statement.<br><br>General guidance is provided below. Consult the submission guidelines for detailed instructions. **Make sure that all information entered here is included in the Methods section of the manuscript.**	This study was approved by the Makerere University School of Medicine Research and Ethics Committee (SOMREC; approval reference: Mak-SOMREC-2025-913) and by Kawempe National Referral Hospital. All participants provided written informed consent before interview. Participants were assigned anonymised codes; no identifying information was included in transcripts or analysis. Participation was voluntary, and participants could withdraw at any time without consequence

**Format for specific study types**<br><br>**Human Subject Research (involving human participants and/or tissue)**<br>• Give the name of the institutional review board or ethics committee that approved the study<br>• Include the approval number and/or a statement indicating approval of this research<br>• Indicate the form of consent obtained (written/oral) or the reason that consent was not obtained (e.g. the data were analyzed anonymously)<br><br>**Animal Research (involving vertebrate animals, embryos or tissues)**<br>• Provide the name of the Institutional Animal Care and Use Committee (IACUC) or other relevant ethics board that reviewed the study protocol, and indicate whether they approved this research or granted a formal waiver of ethical approval<br>• Include an approval number if one was obtained<br>• If the study involved *non-human primates*, add *additional details* about animal welfare and steps taken to ameliorate suffering<br>• If anesthesia, euthanasia, or any kind of animal sacrifice is part of the study, include briefly which substances and/or methods were applied<br><br>**Field Research**<br><br>Include the following details if this study involves the collection of plant, animal, or other materials from a natural setting:<br>• Field permit number<br>• Name of the institution or relevant body that granted permission	
**Data Availability**<br><br>Before publication, Authors are required to make fully available and without restriction all data underlying their findings. Sharing the data underlying your findings increases the visibility, credibility, and impact of your work, while supporting transparency and future scientific discovery. Please see our PLOS Data	All data underlying this study consist of in-depth interview transcripts containing sensitive personal health information shared by participants under assurances of confidentiality. In accordance with PLOS ONE's policy for qualitative research data, representative transcript excerpts supporting all reported themes are included within the paper (Results section). Full transcripts cannot be shared publicly, as doing so would violate participant consent agreements and risk identification of individuals who disclosed stigmatised health experiences. Anonymised thematic data are available from the corresponding author (Kawungu Saad Sessimba; aklatsad@gmail.com) on reasonable request, subject to approval from the Makerere University School of Medicine Research and Ethics Committee (SOMREC; approval reference: Mak-SOMREC-2025-913) and compliance with applicable data protection requirements.

Policy page for detailed information on this policy.<br><br>A **Data Availability Statement**, detailing where the data can be accessed, is required at first submission. Insert your Data Availability Statement in the box below.<br><br>Please see the data reporting section of our submission guidelines for instructions on what you need to include in your Data Availability Statement. Examples of acceptable Data Availability Statements are included here for reference:<br>1. The minimal data set [and accompanying code, where generated] is available at [repository name] via [DOI hyperlink]<br>2. No data was generated by this study. The following existing data sources were used: [dataset name] from [dataset location] available via [web address or DOI hyperlink].<br>3. For Study Protocols: No datasets were generated or analysed during the current study. All relevant data from this study will be made available upon study completion.<br><br>*PLOS allows rare exemptions to address legal and ethical concerns. If you have legal or ethical restrictions, please use the box below to detail these in full sentences for the Journal team to consider.*<br><br>This statement will be typeset if the manuscript is accepted for publication.	

Powered by Editorial Manager® and ProduXion Manager® from Aries Systems Corporation

Manuscript
Click here to access/download;Manuscript;Manuscript-current-submitted.docx

Women's experiences of emergency post-abortion care at Kawempe National Referral Hospital, Uganda: a qualitative phenomenological study

Kawungu Saad Sessimba[1*], Aburi Godfrey James[1], Baluku Andrew[1], Igirimbabazi Pious[1], Kibuuka Balikudembe[1], Keesiga Annette[2‡], Herbert Kayiga[1‡]

[1] Department of Obstetrics and Gynaecology, College of Health Sciences, Makerere University, Kampala, Uganda

[2] Department of Obstetrics and Gynaecology, Kawempe National Referral Hospital, Kampala, Uganda

* Corresponding author – Email: aklatsad@gmail.com (KSS)

ORCID: https://orcid.org/0009-0001-4045-7633

‡ KA and HK are Joint Senior Authors.

Short Title: Women's experiences of emergency post-abortion care at Kawempe National Referral Hospital.

Abstract

Background: Post-abortion care (PAC) encompasses emergency treatment, counselling, contraceptive services, and referral linkages. Emergency post-abortion care (EPAC), the life-saving component of PAC, addresses acute abortion-related complications, including haemorrhage, sepsis, retained products of conception, and severe pain. In Uganda, where abortion is legally restricted and socially stigmatised, women’s care experiences are shaped by clinical

urgency, fear, moral vulnerability, provider interactions, and structural health system constraints. Despite EPAC's centrality to maternal survival, qualitative evidence on how women interpret and evaluate their care experiences in referral hospital settings in Uganda remains limited. This study explored women's experiences of EPAC at Kawempe National Referral Hospital (KNRH) and identified the factors that shaped those experiences.

Methods: A qualitative phenomenological design was employed. Sixteen in-depth interview transcripts from women who received EPAC at KNRH in March–April 2026 were analysed using inductive thematic analysis. The Socio-Ecological Model (SEM) was applied as an interpretive framework.

Results: Six themes were identified: (1) survival and physical relief as the immediate measure of good care; (2) pain, fear, and emotional distress during treatment; (3) reassurance and support as buffers against vulnerability; (4) dignity under pressure: communication and privacy in EPAC; (5) structural barriers across the pathway of care; and (6) experiences beyond discharge: incomplete recovery and uncertainty. Care was frequently evaluated through the lens of survival, yet these accounts co-existed with intense procedural pain, compromised privacy, delays, financial burden, and inadequate post-discharge support. EPAC at KNRH was experienced as a complex encounter shaped by bodily vulnerability, interpersonal dynamics, and system-level constraints.

Conclusions: Strengthening EPAC requires patient-centred approaches that integrate clinical effectiveness with respectful communication, pain management, improved triage, and structured post-discharge support.

Keywords: Emergency post-abortion care; qualitative research; phenomenology; women's experience; reproductive health; Uganda

Introduction

Post-abortion care (PAC) encompasses emergency treatment, counselling, contraceptive services, and referral linkages [1]. Emergency post-abortion care (EPAC), the clinical backbone of PAC, involves urgent management of women presenting with acute abortion-related complications such as haemorrhage, sepsis, retained products of conception, shock, or severe pain [2]. Unlike routine reproductive health services, EPAC is delivered in time-critical contexts where rapid stabilisation takes priority. Yet the quality of care is not determined by technical outcomes alone. Global evidence increasingly recognises that women evaluate care through a broader lens, including communication, dignity, privacy, emotional support, and timeliness [3, 4].

Globally, unsafe abortion accounts for a substantial proportion of maternal morbidity and mortality, predominantly in low- and middle-income countries. Approximately half of all abortions worldwide occur under unsafe conditions, generating complications that require emergency medical intervention [1, 5]. In these settings, EPAC serves as a vital entry point into the health system, often the difference between life and death. However, research consistently shows that survival does not guarantee a positive care experience: women frequently report fear, confusion, lack of information, and perceived mistreatment, even when clinical management is technically effective [6, 7].

In Sub-Saharan Africa (SSA), these challenges are compounded by health system constraints, including limited resources, high patient volumes, and workforce shortages. Women's experiences of post-abortion care across the region are shaped not only by service availability but by the quality of delivery, particularly respectful communication, pain management, and privacy protections [8-10].

In Uganda, the context of EPAC is further shaped by restrictive abortion laws and strong social stigma surrounding abortion and pregnancy loss [11, 12]. Women may delay seeking care due to fear of judgment or legal consequences, and upon presentation, may carry feelings of guilt or shame. These emotional and social dimensions interact with the clinical encounter, shaping how care is experienced, remembered, and evaluated [5, 11].

KNRH, one of Uganda's busiest public maternity facilities, receives a high volume of women with abortion-related emergencies, handling over 200 EPAC cases monthly [13]. Women frequently arrive after having navigated multiple lower-level facilities that lack essential resources such as blood, equipment, or trained personnel [14]. Evidence from related obstetric contexts at KNRH shows that even when clinical care is effective, women may still experience poor communication and emotional neglect [15].

Furthermore, the EPAC experience extends beyond discharge. Women may continue to experience physical symptoms, uncertainty about recovery, concerns about future fertility, and varying levels of social support or stigma within their communities. Previous studies have shown that post-abortion care experiences influence not only immediate recovery but also future health-seeking behaviour and trust in the health system [16, 17].

Despite EPAC's central role in maternal health, most research in Uganda has focused on clinical outcomes, service availability, and utilisation patterns [18-20]. Without evidence on how women themselves interpret and evaluate their care experiences, particularly in emergency contexts, quality improvement efforts risk prioritising technical performance while overlooking the relational, emotional, and structural factors that shape patient experience [21].

This study, therefore, explored women's experiences of EPAC at KNRH through a qualitative lens, applying the Socio-Ecological Model (SEM) to examine factors operating at the individual, interpersonal, facility, and community levels.

Materials and methods

Ethics statement

This study was approved by the Makerere University School of Medicine Research and Ethics Committee (SOMREC; approval reference: Mak-SOMREC-2025-913) and by Kawempe National Referral Hospital. All participants provided written informed consent before interview. Participants were assigned anonymised codes; no identifying information was included in transcripts or analysis. Participation was voluntary, and participants could withdraw at any time without consequence.

Study design

A qualitative phenomenological design was employed. Phenomenology is appropriate when the aim is to understand the lived experiences of individuals who have directly encountered a specific phenomenon [22]. This approach enabled in-depth exploration of the physical, emotional, and social dimensions of women's EPAC experiences, dimensions not accessible through quantitative methods alone.

Study setting

The study was conducted at KNRH in Kampala, Uganda, within the gynaecological emergency unit, where women with abortion-related complications are assessed, managed, and reviewed. KNRH is Uganda's national referral centre for obstetric and gynaecological emergencies, handling over 200 EPAC cases per month (approximately seven cases per day) [13]. EPAC is provided free of charge, with out-of-pocket payments required only for ultrasound scans and medications that are out of stock. Services are available 24 hours a day, 365 days a year, delivered by a multi-cadre team ranging from senior consultants to student health workers.

Study population and data source

The study analysed sixteen in-depth interview transcripts from women who had received EPAC at KNRH. Participants ranged in age from 18 to 45 years and represented diverse socio-demographic and reproductive backgrounds, including variation in marital status, educational attainment, occupation, and parity.

Sampling strategy

Purposive sampling with maximal variation was used to select participants who had direct experience of EPAC and had been discharged within the preceding 14 days. This window allowed sufficient time for initial physical recovery and reflection while minimising recall bias, without approaching women during the acute treatment period. Eligibility required clinical stability at recruitment, informed consent, and willingness to participate in an interview conducted in English or Luganda.

Sampling was guided by the principle of data saturation: data collection continued until no new codes, categories, or themes emerged from additional transcripts [23]. Saturation was evident as codes became consistent and repetitive across the later transcripts, confirming analytical adequacy.

Data collection

Data were collected through semi-structured in-depth interviews following a two-stage recruitment process. In the first stage, women who had received EPAC were identified at discharge; their contact details were obtained, and a scheduled review appointment was confirmed. In the second stage, on or around the review date, the research team confirmed participants' arrival at the hospital. Women who met eligibility criteria were approached by a trained research assistant, given a verbal explanation of the study, and offered time to consider participation before written informed consent was obtained.

Of the women approached during the study period (March–April 2026), eight declined to participate citing time constraints or personal concerns. The sixteen who consented were interviewed individually in a consultation room at the gynaecological emergency unit, prioritising privacy. The interview guide covered: pathway to care and first presentation; reception, triage, and waiting; treatment and procedural experience; privacy and dignity; provider communication and information-giving; pain and emotional responses; social support; challenges during and after treatment; and post-discharge recovery. Interviews were conducted in English or Luganda according to participant preference, audio-recorded, and transcribed verbatim. Back-translation procedures were applied where applicable.

Data analysis

Transcripts were read repeatedly to develop familiarity with the data. Meaningful units of text were identified, coded, and grouped into categories based on similarities and patterns across accounts. Themes were generated inductively through thematic analysis, moving iteratively between participants' language and broader interpretive concepts. Constant comparison across transcripts was used to identify both common patterns and divergent experiences [23].

The SEM was applied as an interpretive lens at later stages of analysis to organise findings by the level at which they operated: individual, interpersonal, facility, and community. This framework was originally developed to explain how human experiences are shaped across multiple nested levels of influence [24] and has since been applied to health service research to guide multi-level interpretive frameworks [25]. This approach, of using the SEM as an organising framework rather than a deductive coding template, allowed themes to remain grounded in participants' narratives while being situated within a theoretically coherent multi-level account.

Rigour and trustworthiness

Rigour was ensured by applying Lincoln and Guba's criteria for trustworthiness: credibility, dependability, and transferability [22, 26]. Credibility was enhanced through immersive, iterative engagement with participants' own words. Dependability was ensured through consistent application of the coding framework and systematic documentation of analytical decisions. Transferability was supported by detailed descriptions of the study setting, participant characteristics, and analytical process. Transparency was maintained by attending to both positive

and negative experiences, including contradictions within individual accounts, to avoid selective reporting.

Results

Participant characteristics

Sixteen in-depth interview transcripts were included in the final analysis. Participants ranged in age from 18 to 45 years and represented diverse social and reproductive backgrounds.

Table *1* presents socio-demographic characteristics.

Table 1. Socio-demographic characteristics of study participants (March–April 2026, n=16).

Characteristic	Category	Frequency (n=16)
**Age (years)**	18–24	3
	25–34	7
	35–45	6
**Marital status**	Married	9
	Single/unmarried	7
**Education level**	Primary	4
	Lower secondary	6
	Upper secondary	4
	Post-secondary	2
**Occupation**	Housewife/unemployed	6
	Informal employment	7
	Formal employment	3
**Number of children**	0	4
	1–2	6
	3–4	4
	5+	2

Overview of themes

Table 2 summaries all the themes, sub-themes and corresponding meaningful units/codes.

Table 2. Overview of themes, sub-themes, and meaningful units/codes

Theme	Sub-theme	Meaningful units (codes)
**1. Survival and physical relief as immediate measure of good care**	Physical stabilisation and symptom relief	Bleeding stopped; pain reduced; felt saved
	Rapid emergency response	Attended quickly; prioritised; emergency action taken
	Survival as minimum threshold	Gratitude for survival; care judged by outcome
**2. Pain, fear, and emotional distress during treatment**	Procedural pain and bodily suffering	Painful evacuation; severe discomfort; helplessness
	Fear of death and acute uncertainty	Fear of dying; shock; severe bleeding; anxiety
	Emotional conflict and self-judgment	Guilt; regret; self-blame; internal distress
**3. Reassurance and support as buffers against vulnerability**	Provider reassurance and emotional calming	Comfort; counselling; encouragement; calming words
	Family and social support	Transport; money; caregiving; relative presence
	Absence of support	Alone; abandonment; dependence; emotional isolation
**4. Dignity under pressure: communication and privacy in EPAC**	Respectful communication and explanation	Welcomed; informed; listened to; spoken to well
	Dismissive or judgmental communication	Rude language; blame; harsh tone; not listened to
	Privacy preserved or violated	Curtain used; exposure; embarrassment; many observers
**5. Structural barriers across the pathway of care**	Delays and waiting under emergency conditions	Long waits; delayed review; bleeding while waiting
	Overcrowding and limited resources	Many patients; lack of blood; staff shortage
	Financial burden within care	Paying for scan; buying medicines; borrowing money
	Referral and transfer challenges	Moved between facilities; no blood; delayed care
**6. Experiences beyond discharge: incomplete recovery and uncertainty**	Persistent symptoms after discharge	Ongoing pain; bleeding; weakness; possible infection
	Limited follow-up and continuity gaps	No review; no follow-up call; no check-up
	Fertility and reproductive uncertainty	Future pregnancy concerns; fear about fertility
	Social support, silence, and stigma after discharge	Selective disclosure; fear of judgment

Theme 1: Survival and physical relief as the immediate measure of good care

For most women, the first and most immediate meaning of EPAC was survival. Participants described care as "good" when it produced recognisable bodily improvement such as relief from pain, reduced bleeding, or a sense that the condition had been controlled in time. Clinical recovery formed the earliest and strongest basis for evaluating care.

> *"They worked on me very well... they focused on me being fine. The doctors kept checking on me, and when I was told I could go home, I felt like a different person from the one who had come in. I was really happy with the way they treated me." (IDI_16, 23 years)*

> *"When I went to the hospital, I was feeling lower abdominal pain with every step I took, but after removing the pregnancy-related items from inside me, I felt relieved from the pain. It was like something heavy had been removed from inside me." (IDI_6, 28 years)*

The speed of response also shaped experience. Visible urgency from providers conveyed both seriousness and competence, reinforcing women's confidence that care was effective.

> *"When I reached there, they all rushed, wanting to know what had happened to me. One doctor was already asking questions before I had even sat down, and another was already preparing to examine me. That reaction made me feel that my situation was being taken seriously." (IDI_14, 42 years)*

However, positive accounts were not uncomplicated. Women who described care as good still frequently narrated pain, fear, or emotional trauma within the same account, illustrating that survival, in the context of acute emergency, may temporarily overshadow shortcomings in communication, privacy, or emotional support.

Theme 2: Pain, fear, and emotional distress during treatment

Despite the primacy of survival, women also described EPAC as intensely painful and emotionally distressing. Procedural pain emerged strongly, particularly during uterine evacuation.

> *"The moment when the machine was pulling the placenta out was so painful. It reached the point that when I heard metals being banged, I got scared and started shaking. I kept asking them to stop, but they told me to be patient because they were almost done." (IDI_3, 32 years)*

> *"The pain was too much when the doctor put in the hand, it was like my whole inside was being pulled out. I cried and asked him to stop, but he said he had to finish what he was doing. I sometimes dream of what he did to me, and I wake up feeling that same pain all over again." (IDI_4, 21 years)*

Fear of death was a recurring feature. Women who arrived haemorrhaging or acutely unwell often described uncertainty about what was happening within their bodies, intensifying fear and making treatment emotionally overwhelming.

> *"I thought I was going to die because of how much blood I had lost before reaching the hospital. But there was a female doctor who told me that people had come in worse conditions than mine and had survived. That calmed me a little, even though the fear was still there." (IDI_16, 23 years)*

Beyond physical suffering, some women described emotional conflict linked to the pregnancy event itself. Feelings of guilt, regret, or self-blame emerged across several transcripts, extending the meaning of distress beyond the procedure to include internal moral struggle. Women also expressed anxiety about future fertility.

> *"My question was, if I want to conceive, what is the right period for me to get pregnant? They gave me a range, saying six months, eight months, but I wanted the exact answer because I do not want to make any mistakes. Not knowing the right time kept disturbing my mind even after I had left the hospital." (IDI_15, 38 years)*

Theme 3: Reassurance and support as buffers against vulnerability

Support from healthcare providers, family, and social networks shaped women's capacity to endure pain and fear. Provider reassurance emerged as a particularly important form of emotional support, functioning as a therapeutic intervention in its own right.

> *"The doctor took time and counselled me and comforted me, making me feel calm. He told me I would be fine, since people in worse conditions had survived and gone back to live their normal lives... I had arrived at the hospital believing I was going to die, and after he spoke to me, I started to think that maybe I would make it." (IDI_2, 45 years)*

Family support also played a major role, enabling women to access treatment, meet unexpected costs, and cope during recovery.

> *"My husband helped me throughout the whole time I was in the hospital. Whenever I needed a blood transfusion, he would provide the money, and if he didn't have it, he would borrow from relatives or friends without complaining. Knowing he was there and willing to do whatever was needed gave me the strength to go through everything." (IDI_2, 45 years)*

Not all women had such support. Those who arrived alone described intensified vulnerability and complete dependence on staff.

> *"I had come alone because I did not want anyone to know why I was going to the hospital. But when I got there, and things became serious, I realised how much I needed someone. I couldn't do anything on my own, and I had to depend completely on the nurses and doctors for everything. I was helpless..." (IDI_1, 20 years)*

Theme 4: Dignity under pressure — communication and privacy in EPAC

Women's experiences were significantly influenced by whether they felt treated with dignity. Dignity was expressed through the quality of communication and the protection of physical privacy.

> *"He didn't talk badly to me at all; he asked me what was wrong and even took me away from the crowded reception because I was in pain and began examining me immediately. When I asked him about what he thinks about my condition, he explained until I understood, which I did not expect from a government hospital." (IDI_11, 28 years)*

However, other women experienced communication as dismissive or judgmental. In the context of abortion-related complications, such communication carried moral weight, deepening emotional discomfort.

> *"The doctor kept asking why I was alone and why we got pregnant at such a young age, and the way he was asking made me feel like I had done something very wrong. I wanted to explain myself, but he did not seem willing to listen. That made me feel ashamed on top of everything else I was already going through." (IDI_1, 20 years)*

Privacy was conditional: some women described curtains and restricted visibility; others reported feeling exposed during procedures.

> *"They worked on me in the open when everyone was seeing, there was no curtain and no screen between the other people in the room and me. I just felt embarrassed and kept looking away, hoping the people nearby were not watching... I had to endure it because I needed the treatment." (IDI_1, 20 years)*

Theme 5: Structural barriers across the pathway of care

Women's experiences were strongly shaped by structural conditions: delays, overcrowding, resource shortages, financial burden, and repeated movement across facilities before reaching definitive care.

> *"I waited for over 4 hours, and no doctor had checked on me, yet I was bleeding so much that I had to keep changing my clothes. Doctors were passing me by and not really listening when I tried to tell them how bad things were... We just sat there, I and two other women who had also been waiting a long time, all of us suffering in silence." (IDI_10, 26 years)*

Delays were not merely logistical; they became part of the emotional experience of illness, increasing fear and the feeling of neglect.

> *"If you don't fight for your life, you can wait till evening without anyone bothering to check on you. I had to stand up twice and go and ask about my situation before they finally attended to me... not everyone has the strength to keep asking to be helped." (IDI_12, 25 years)*

Financial burden was a major issue. Women reported paying for scans and medications at a public facility, adding stress and uncertainty to emergency care.

"The doctor asked for money for the scan, it was sh.20,000, but I had only sh.15,000, and I didn't know what to do. I was sick and worried and now I had to think about money on top of everything else..." (IDI_6, 28 years)

Many women's experiences began before arrival at KNRH, shaped by referral pathways involving multiple facilities.

"They did not have blood to put on me at the first hospital, and yet I was losing so much that the doctor said I needed to get blood urgently. They told my husband to take me somewhere else... By the time I finally got the blood I needed, I had been moving from place to place for hours while getting weaker." (IDI_2, 45 years)

Theme 6: Experiences beyond discharge — incomplete recovery and uncertainty

Women's experiences of EPAC did not end at discharge. Persistent physical symptoms, limited follow-up, unanswered questions, and variable social support shaped how they understood their recovery.

"Right now, I am feeling some pain, a little pain in my lower abdomen that has not gone away since I left the hospital. I also think I keep producing pus because there is something that comes out which does not look normal to me... nobody told me what to expect after going home." (IDI_6, 28 years)

Follow-up care was largely absent, contributing to uncertainty about what was normal and when to seek help.

> *"No one has called me since I was discharged, not to check if I am okay or if the symptoms have changed. I thought maybe they would follow up in a day or two, but nothing came of it. After you're discharged, that's it." (IDI_6, 28 years)*

Social context also mattered after discharge. Some women limited disclosure due to stigma, meaning that recovery was not only medical but also social and emotional.

> *"My mum called me, and I told her I was fine, because I did not want her to worry about the details of what had happened... I was not ready for what she would think of me..." (IDI_13, 34 years)*

Discussion

This study explored women's experiences of EPAC at KNRH, Uganda, and identified the multi-level factors shaping those experiences. The findings reveal that women evaluated EPAC primarily through survival, a fear-driven reordering of perception in which physical rescue temporarily overshadowed all other quality dimensions. This pattern is consistent with earlier qualitative work [6, 7] and extends it by demonstrating that the primacy of survival in emergency settings was not merely a preference but a response to acute mortal threat. Importantly, this challenges the sole use of patient satisfaction surveys as quality indicators in EPAC, since women who survived life-threatening complications often rated care positively regardless of whether communication, pain management, or privacy were adequate [3, 4].

Once the immediate danger subsided, the embodied suffering that accompanied treatment came into sharper perspective. Pain in EPAC was not only a clinical symptom but it was an interpretive experience carrying social, moral, and psychological weight. This convergence of physical and

emotional suffering, documented by Coast et al. [27] and Ganatra et al. [5], was extended here into an emergency context, revealing that inadequate pain management amplified distress rather than containing it.

Reassurance from providers mediated between suffering and the capacity to endure it, functioning as a therapeutic intervention. When providers spoke calmly, explained what was happening, or remained emotionally present during painful procedures, women reported better ability to manage fear. This was consistent with Baum et al. [6, 28], Whitehouse et al. [29], and Chekol et al. [30]. In emergency settings, this function was amplified since women with severe haemorrhage were far more dependent on reassurance than those undergoing planned procedures. Absence of family support intensified this dependence [8, 16, 31].

In Uganda's context of legal restriction and stigma, judgmental communication carried moral weight beyond poor service quality. It signalled condemnation to an already vulnerable woman. Cleeve [12] and Ouedraogo et al. [7] documented similar patterns; this study extended both findings by showing that in emergency settings where women cannot leave to seek care elsewhere, such communication becomes coercive. Privacy violations compounded this by creating real social exposure risks in communities where abortion-related care is stigmatised.

Structural conditions constrained provider capacity for dignified care. Delays, shortages, and out-of-pocket costs at a public facility shaped the conditions under which relational care was possible which was consistent with prior evidence [9, 14, 18]. Fragmented referral, documented by Ouma et al. [32], meant that for many women, EPAC had begun hours earlier under deteriorating conditions before reaching KNRH.

Discharge marked not the end of EPAC but a transition to largely unstructured recovery. Most prior Ugandan studies examined access and utilisation, not the post-discharge period [18-20]. Lythgoe et al. [16, 17] showed that post-discharge experiences in SSA shaped future engagement with health services. This study confirmed and extended that finding specifically for EPAC: fertility-related uncertainty that persisted when discharge counselling failed to address conception timing constituted a psychological extension of the care experience itself.

Stillman et al. [33] showed that postabortion care experiences in Uganda were characterised by both physical suffering and emotional distress, consistent with findings from this study. Kabunga et al. [34] highlighted the added complexity for women living with HIV in northern Uganda, where fear, stigma, and moral conflict overlapped with the physical dimensions of care, extending the psychological meaning of EPAC well beyond the clinical encounter.

Factors that influenced the women's experiences guided by the SEM

Applying the SEM as an interpretive framework revealed that no single level fully accounted for women's experiences; findings at each level were necessary but not sufficient.

At the individual level, bodily vulnerability, such as severity of haemorrhage, pain intensity, fear of death, and reproductive anxiety, shaped how women entered the care encounter and what they were able to perceive and evaluate. Women in extreme clinical distress were least able to assert preferences or notice deficiencies, and were most dependent on provider behaviour.

At the interpersonal level, provider communication, family support, and provider attitudes were decisive mediating factors, themselves shaped by facility-level conditions (overcrowding, staff pressures) and community-level factors (stigma). Cleeve [12] showed that provider stigma in

Kampala shaped interactions with women seeking post-abortion care; this study extended that finding by tracing how stigma translated into specific communication practices that deepened women's distress.

At the facility level, KNRH's structural conditions, such as high patient volumes, blood product shortages, inadequate privacy arrangements, and absent follow-up systems, shaped the relational possibilities of care beyond its technical delivery, consistent with SSA-wide evidence [9, 18].

At the community level, abortion stigma in Uganda shaped the entire care trajectory: from delayed care-seeking, through morally loaded interpretations of provider behaviour, to post-discharge silence and selective disclosure. Ouma et al. [32] and Ndayisenga et al. [35] documented how stigma shapes abortion care pathways across East Africa; this study found that stigma operated within the facility during care and continued after discharge during recovery.

The SEM resolved an apparent paradox: how women could simultaneously describe care as life-saving and inadequate. Survival was secured at the individual and facility levels through clinical intervention; dignity was undermined at the interpersonal level by poor communication and at the facility level by compromised privacy and delays; and community-level stigma amplified these indignities. The SEM demonstrated that EPAC quality was not a single outcome but a product of multiple, interacting, and sometimes contradictory forces operating simultaneously from crisis to post-discharge recovery.

Strengths and limitations

This study is among the few qualitative investigations focused specifically on women's experiences of EPAC at the national referral hospital level in Uganda, providing evidence directly

relevant to policy and practice in a high-volume, high-acuity setting. The use of in-depth interview data and thematic analysis captured the depth, complexity, and contextual meaning of women's experiences beyond what patient satisfaction surveys can access. Application of the SEM enabled a multi-level analysis that went beyond describing what women experienced to explaining the factors and interactions that produced those experiences. Inclusion of sixteen participants with diverse socio-demographic characteristics strengthened the analytical breadth of the findings.

The main limitation is that the study was conducted at a single site. All participants received care at KNRH, whose institutional characteristics such as high volume, referral context, specific staffing patterns, may not transfer directly to lower-level facilities, private-sector settings, or rural hospitals [21]. There is also potential for social desirability bias in interviews about care quality; however, the presence of multiple negative accounts across the dataset suggests participants felt sufficiently safe to share critical experiences. The 14-day post-discharge recruitment window may have been too short to capture the full psychological consequences of fertility uncertainty and emotional processing of guilt or grief, which tend to become more salient over longer periods. Finally, data were collected across a single two-month period at one facility; conditions prevailing during that period such as stock availability, staffing, and patient flow, may not represent the full range of conditions over time. Longitudinal follow-up would provide a more complete picture of recovery trajectories and lasting dimensions of the care experience.

Conclusions

EPAC at KNRH was experienced not as a single clinical event but as a multi-dimensional encounter shaped by bodily vulnerability, provider behaviour, structural conditions, and the social context of abortion. Survival was necessary but not sufficient as a measure of quality.

Communication, pain management, and privacy were core quality dimensions, not supplementary comforts. Structural barriers including delays, shortages, out-of-pocket costs, and fragmented referrals constrained dignified care. The experience of EPAC extended beyond discharge, with recovery largely unstructured and fertility uncertainty persisting without follow-up.

Improving the quality of EPAC requires a patient-centred approach that integrates clinical effectiveness with respectful, non-judgmental communication, systematic pain management, improved triage, better use of privacy measures, reliable availability of essential medicines and blood products, stronger referral coordination, and structured discharge counselling and follow-up. Provider sensitisation should address stigma as a direct determinant of care quality. Future research should examine EPAC experiences across the full referral pathway, include provider perspectives, and assess long-term physical, psychological, and reproductive outcomes after emergency care.

Author contributions

Conceptualisation: KSS, HK, KA

Data curation: KSS, AGJ, BA, IP, KB

Formal analysis: KSS

Investigation: KSS, AGJ, BA, IP, KB

Methodology: KSS, HK, KA

Project administration: KSS, AGJ, BA, IP, KB

Resources: KSS, AGJ, BA, IP, KB

Supervision: HK, KA

Validation: HK, KA

Visualization: KSS

Writing – original draft preparation: KSS

Writing – review and editing: KSS, HK, KA

Competing interests

The authors declare that they have no competing interests.

Data availability statement

All data underlying this study consist of in-depth interview transcripts containing sensitive personal health information shared by participants under assurances of confidentiality. In accordance with PLOS ONE's policy for qualitative research data, representative transcript excerpts supporting all reported themes are included within the paper (Results section). Full transcripts cannot be shared publicly, as doing so would violate participant consent agreements and risk identification of individuals who disclosed stigmatised health experiences. Anonymised thematic data are available from the corresponding author (Kawungu Saad Sessimba; aklatsad@gmail.com) on reasonable request, subject to approval from the Makerere University School of Medicine Research and Ethics Committee (SOMREC; approval reference: Mak-SOMREC-2025-913) and compliance with applicable data protection requirements.

Acknowledgments

The authors thank the women who shared their experiences despite the emotional and physical difficulties they had undergone. Their voices made this work possible. We acknowledge Kawempe

National Referral Hospital for providing the research setting. We thank the research assistants who supported data collection and the Department of Obstetrics and Gynaecology, Makerere University, for academic mentorship throughout this work.

References

1. World Health Organization. Abortion care guideline. Geneva: WHO; 2022. Available from: https://www.who.int/publications/i/item/9789240039483
2. Bridwell RE, Carius BM, Mason J, Long B, Koyfman A. Post-abortion complications: a narrative review for emergency clinicians. West J Emerg Med. 2022;23(6):919–925. doi: 10.5811/westjem.2022.8.57929
3. Kruk ME, Leslie HH. Quality of basic maternal care functions in low-income countries — Authors' reply. Lancet Glob Health. 2017;5(5):e486. doi: 10.1016/S2214-109X(17)30148-1
4. World Health Organization. Quality, equity, dignity: a network for improving quality of care for maternal, newborn and child health. Geneva: World Health Organization; 2017.
5. Ganatra B, Nguyen TT, Rowe H, Kapp N, Cameron S, Baraitser P, et al. Women's experiences of facility-based abortion care: a WHO qualitative evidence synthesis. Soc Sci Med. 2025;365:117564. doi: 10.1016/j.socscimed.2024.117564
6. Baum SE, White K, Hopkins K, Grossman D, Potter JE. Abortion quality of care from the client perspective: a qualitative study in India and Kenya. Health Policy Plan. 2021;36(9):1362–1370. doi: 10.1093/heapol/czab065
7. Ouedraogo R, Odera D, Ochieng C, Okal J, Kim C, Obare F. "They talked to me rudely". Women perspectives on quality of post-abortion care in public health facilities in Kenya. Reprod Health. 2023;20(1):57. doi: 10.1186/s12978-023-01580-5.

8. Mutua MM, Kimani S, Ettarh R, Amendah DD. Factors associated with delays in seeking post abortion care among women in Kenya. BMC Pregnancy Childbirth. 2015;15:200. doi: 10.1186/s12884-015-0660-7

9. Muga W, Magoke-Mhoja S, Odeny L, Mbuyita S, Kaliti S, Banda J. Barriers to post-abortion care service provision: a cross-sectional analysis in Burkina Faso, Kenya and Nigeria. PLOS Glob Public Health. 2024;4(3):e0001862. doi: 10.1371/journal.pgph.0001862.

10. Kumah E. Patient experience of care in sub-Saharan Africa: a narrative overview. Int J Healthc Manag. 2025;18(1):38–45. doi: 10.1080/20479700.2023.2265666

11. Paul M, Gemzell-Danielsson K, Kiggundu C, Kiguli J, Mirembe F. Barriers and facilitators in the provision of post-abortion care at district level in central Uganda — a qualitative study focusing on task sharing between physicians and midwives. BMC Health Serv Res. 2014;14:28. doi: 10.1186/1472-6963-14-28

12. Cleeve A. Post-abortion care in Uganda: improving access and quality of care through task sharing and exploring the perspectives of young women and healthcare providers [dissertation]. Karolinska Institutet; 2019. Available from:https://openarchive.ki.se/xmlui/handle/10616/46753

13. Kawempe National Referral Hospital. KNRH PAC register. Kampala: Kawempe National Referral Hospital; 2025.

14. Atuhairwe S, Denno DM, Byamugisha J, Winikoff B, Gemzell-Danielsson K. Abortion-related near-miss morbidity and mortality in 43 health facilities with differences in readiness to provide abortion care in Uganda. BMJ Glob Health. 2021;6(2):e003274. doi: 10.1136/bmjgh-2020-003274

15. LoPoni CT, Muwanguzi-Kizza D, Nandawula E, Byamugisha J. "They just told me to go to theatre": women's experiences and support needs following emergency caesarean section in Kawempe National Referral Hospital, Uganda. BMC Pregnancy Childbirth. 2025;25(1):1–13. doi: 10.1186/s12884-025-07833-4.

16. Lythgoe C, Middleton T, Regan M, Martin G, Doyle T, Mackay D. How women's experiences and perceptions of care influence uptake of postnatal care across sub-Saharan Africa: a qualitative systematic review. BMC Pregnancy Childbirth. 2021;21(1):485. doi: 10.1186/s12884-021-03910-6

17. Udho S, Nabirye RC, Atuyambe L, Turinawe E, Muhwezi WW. Person-centered maternity care and satisfaction with post-abortion care: a facility-based cross-sectional survey in Northern Uganda. Womens Health. 2025;21:17455057251318897. doi: 10.1177/17455057251318897

18. Izugbara C, Wekesah F, Egesa CP. Availability, accessibility and utilization of post-abortion care in Sub-Saharan Africa: a systematic review. Health Care Women Int. 2020;41(7):732–760. doi: 10.1080/07399332.2019.1703991

19. Jackline A, Opio G, Okello S, Anguzu R, Muyindike WR, Bbosa GS. Exploring the utilization of postabortion care services and related factors among women at a tertiary health facility in Gulu, Northern Uganda. Women's Health. 2024;20:17455057241295896. doi: 10.1177/17455057241295896

20. Anena E, Auma AG. Factors post abortion care utilization and associated factors among women of reproductive age in Lira City: a cross-sectional study. Stud J Health Res Afr. 2025;6(9):11. doi: 10.51168/sjhrafrica.v6i9.1908

21. Oluka S. Patients' satisfaction with post-abortion care services in Mulago Hospital: Makerere University College of Health Sciences; 2014. Available from: http://hdl.handle.net/10570/4055

22. Creswell JW, Poth CN. Qualitative inquiry and research design: choosing among five approaches. 4th ed. Thousand Oaks: SAGE; 2018.

23. Guest G, Namey E, Chen M. A simple method to assess and report thematic saturation in qualitative research. PLoS ONE. 2020;15(5):e0232076. doi: 10.1371/journal.pone.0232076

24. Stokols D. Translating social ecological theory into guidelines for community health promotion. Am J Health Promot. 1996;10(4):282–98. doi: 10.4278/0890-1171-10.4.282

25. Scholmerich VLN, Kawachi I. Translating the Socio-Ecological Perspective Into Multilevel Interventions: Gaps Between Theory and Practice. Health Educ Behav. 2016;43(1):17–20. doi: 10.1177/1090198115605309

26. Morse JM. Critical analysis of strategies for determining rigor in qualitative inquiry. Qual Health Res. 2015;25(9):1212–1222. doi: 10.1177/1049732315588501

27. Coast E, Norris AH, Moore AM, Freeman E. Abortion and well-being: a narrative literature review. SSM Qual Res Health. 2025;7:100508. doi: 10.1016/j.ssmqr.2024.100508

28. Baum SE, Andipatin MG, Foster AM, Gerdts C. Quality of care from the perspective of people obtaining abortion: a qualitative study in four countries. BMJ Open. 2023;13(9):e067513. doi: 10.1136/bmjopen-2022-067513

29. Whitehouse KC, Kim CR, Ganatra B, Blumenthal PD, Tuncalp O, Hindocha C, et al. Perceptions of quality in abortion care in England and Wales. Reprod Health. 2021;18(1):134. doi: 10.1186/s12978-021-01270-0

30. Chekol BM, Fentie AM, Dagnaw FT, Mekonnen CK, Gebeyehu NA, Endalew E. Person-centered abortion care in public health facilities across four regions of Ethiopia: a cross-sectional quantitative study of client experiences. Front Reprod Health. 2024;6:1331682. doi: 10.3389/frph.2024.1331682

31. Baynes C, Baffico A, Nowicka M, Lema C, Likindikoki S, Ngilangwa DP. Women's satisfaction with and perceptions of the quality of postabortion care at public-sector facilities in mainland Tanzania and Zanzibar. Glob Health Sci Pract. 2019;7(Suppl 2):S299–S314. doi: 10.9745/GHSP-D-19-00026

32. Ouma OJ, Kabiru CW, Musembi P, Mutua M. Pathways to medical abortion self-use (MASU): results from a cross-sectional survey of women's experiences in Kenya and Uganda. BMC Women's Health. 2023;23(1):412. doi: 10.1186/s12905-023-02570-2

33. Stillman M, Karp C, Atuyambe L, Kibira S, Ouma OJ. Postabortion and safe abortion care coverage, capacity, and caseloads during the global gag rule policy period in Ethiopia and Uganda. BMC Health Serv Res. 2023;23(1):127. doi: 10.1186/s12913-022-09017-8

34. Kabunga A, Nankinga Z, Auma AG, Opollo MS, Wafula ST. Phenomenology of induced abortion in Northern Uganda among HIV-positive women following an unintended pregnancy. Open Access J Contracept. 2023;14:73–82. doi: 10.2147/oajc.s407547

35. Ndayisenga JP, Semasaka Sengoma JP, Mbarushimana V, Luzige S, Mulindwa F. Factors influencing abortion care-seeking outside of formal healthcare settings: lived experiences from Rwandan young women. BMC Women's Health. 2024;24(1):170. doi: 10.1186/s12905-024-03410-7

Supporting information

S1 Interview guide: for women who have received health care for management of abortion related complications at KNRH-English version

S2 COREQ Checklist. COREQ (Consolidated Criteria for Reporting Qualitative Research) checklist for the study.

Supporting Information

Click here to access/download
Supporting Information
S1 File.pdf

Click here to access/download
Supporting Information
S2 File.pdf
