# Supplementary material for: Women’s experiences of emergency post-abortion care at Kawempe National Referral Hospital, Uganda: A qualitative descriptive study
Source: PLoS One. 2026 Nov 5;21(11):e0357615. doi: 10.1371/journal.pone.0357615 (PMC13739178; doi:10.1371/journal.pone.0357615)
Supplement: Supplementary file 3 [file pone.0357615.s002.docx]

**S2 File_CONSOLIDATED CRITERIA FOR REPORTING QUALITATIVE RESEARCH (COREQ)**

**Manuscript:** *Women's experiences of emergency post-abortion care at Kawempe National Referral Hospital, Uganda: a qualitative descriptive study*

**Authors: Kawungu Saad Sessimba¹*, Aburi Godfrey James¹, Baluku Andrew¹, Igirimbabazi Pious¹, Kibuuka Balikuddembe¹, Keesiga Annette²‡, Herbert Kayiga¹‡**

**Target journal:** PLOS ONE | Ethics: SOMREC, Mak-SOMREC-2025-913

| **No.** | **Item** | **Guide question / description** | **Response** | **Reported on Page(s)** |
| --- | --- | --- | --- | --- |
| **DOMAIN 1: Research team and reflexivity** | | | | |
| ***Personal characteristics*** | | | | |
| **1** | Interviewer / facilitator | Which author/s conducted the interview or focus group? | In-depth interviews were conducted by the principal investigator (Kawungu Saad Sessimba, MBChB) supported by trained research assistants who facilitated participant approach, consent, and data collection. | Pages 8, 12 |
| **2** | Credentials | What were the researcher's credentials, e.g. PhD, MD? | Principal investigator: MBChB (Makerere University), postgraduate candidate in MMed Obstetrics and Gynecology. Supervisors (Herbert Kayiga: MBChB, MMed, MPH, PhD; Keesiga Annette: MBChB, MMed, MPH) stated on cover page and title page. | Not reported |
| **3** | Occupation | What was their occupation at the time of the study? | Principal investigator: postgraduate student, Department of Obstetrics and Gynecology, CHS, Makerere University. Research assistants: registered midwives at KNRH. | Page 1 |
| **4** | Gender | Was the researcher male or female? | Not explicitly stated in the manuscript. The researcher is however male. | Not reported |
| **5** | Experience and training | What methodological experience and training did the researcher have? | The principal investigator is enrolled in a postgraduate research degree. Research assistants underwent standardized training in study protocol, informed consent, and semi-structured interviewing. Formal methodological qualifications not detailed beyond the degree program. | Pages 7, 12 |
| ***Relationship with participants*** | | | | |
| **6** | Relationship established | Was a relationship established prior to study commencement? | No prior relationship. Two-stage approach: women were identified at discharge (Stage 1); the research team approached them at the scheduled follow-up review visit (Stage 2). First contact for study purposes occurred during the discharge stage. | Pages 8, 12 |
| **7** | Participant knowledge of the interviewer | What did participants know about the researcher e.g. personal goals, reasons for doing the research? | Before consent was obtained, each participant was given a verbal explanation of the study purpose, the researcher's role as a postgraduate student, and the voluntary nature of participation with no effect on clinical care. | Pages 8, 12 |
| **8** | Interviewer characteristics | What characteristics were reported about the interviewer/facilitator? | Research assistants were trained registered midwives familiar with KNRH's clinical environment and able to conduct interviews in English and Luganda. Interviews were conducted individually in a private consultation room. | Pages 8, 12–13 |
| **DOMAIN 2: Study design** | | | | |
| ***Theoretical framework*** | | | | |
| **9** | Methodological orientation and theory | What methodological orientation was stated to underpin the study, e.g. grounded theory, discourse analysis, ethnography, and phenomenology? | Qualitative descriptive design, without commitment to a specific phenomenological, grounded-theory, or narrative analytic tradition. Inductive thematic analysis was used for coding and theme generation. The Socio-Ecological Model (SEM) was applied as an organising interpretive framework at later stages of analysis to situate findings across individual, interpersonal, facility, and community levels. | Pages 7, 13–14 |
| ***Participant selection*** | | | | |
| **10** | Sampling | How were participants selected, e.g. purposive, convenience, consecutive, snowball? | Purposive sampling with maximal variation strategy, ensuring diversity in age, marital status, educational attainment, occupation, and parity to maximize analytical breadth. | Page 11 |
| **11** | Method of approach | How were the participants approached? E.g. face to face, telephone, mail | Two-stage face-to-face approach: (1) at discharge from KNRH, eligible women were identified and a review appointment confirmed; (2) at the review visit, the research assistant approached participants face to face, provided a verbal briefing, and obtained written informed consent. | Page 12 |
| **12** | Sample size | How many participants were in the study? | Sixteen (16) participants: the women who consented among 24 approached during the two-month recruitment window (8 declined). Sample size was guided by thematic saturation, judged narratively as fieldwork and analysis ran side by side, not by an a priori power calculation. | Pages 11–12, 16 |
| **13** | Non-participation | How many people refused to participate or dropped out? Reasons? | Eight women declined participation during March–April 2026, citing time constraints or personal concerns. No participants withdrew after enrolment. No dropouts occurred. | Pages 11–13 |
| ***Setting*** | | | | |
| **14** | Setting of data collection | Where was the data collected? E.g. home, clinic, workplace | A private consultation room within the gynecological emergency unit, KNRH, Kampala, Uganda. Setting was chosen to ensure privacy and minimize disruption to clinical care. | Pages 9, 13 |
| **15** | Presence of non-participants | Was anyone else present besides the participants and researchers? | Not explicitly reported. Interviews were conducted individually in a private room, indicating no other patients or staff were present during data collection. | Page 13 |
| **16** | Description of sample | What are the important characteristics of the sample, e.g. demographic data, date? | Participants aged 18–45 years. Table 1 reports socio-demographic and clinical characteristics: age, marital status, education, occupation, number of children, gestational age, presenting complication, treatment received, transfusion need, and referral status. All participants were interviewed at least 14 days after discharge (Sampling strategy; Data collection). Data collected March–April 2026. | Pages 10, 12, 16 |
| ***Data collection*** | | | | |
| **17** | Interview guide | Were questions, prompts, guides provided by the authors? Was it pilot tested? | Semi-structured interview guide used, opening with an account of the participant's overall experience of care, including probes on privacy, confidentiality, and feeling respected, and continuing with: prior knowledge and expectations of care; interactions with health workers; communication and information-sharing; support from family and others; community attitudes and stigma; recommendations for the health system and policy; and a final open reflection. Guide provided as S1 File. Pilot testing was done. | Page 13 |
| **18** | Repeat interviews | Were repeat interviews carried out? If yes, how many? | Not applicable. Each participant was interviewed once. | N/A |
| **19** | Audio/visual recording | Did the research use audio or visual recording to collect the data? | Yes. Interviews were audio-recorded with participant consent and transcribed verbatim. Back-translation procedures were applied where interviews were conducted in Luganda. | Page 13 |
| **20** | Field notes | Were field notes made during and/or after the interview? | Not reported as field notes. Each interviewer kept a reflexive journal entry after every interview covering the interview setting, the participant's emotional state, and the interviewer's own reactions (Researcher positionality). | Pages 8–9 |
| **21** | Duration | What was the duration of the interviews or focus group? | Interviews lasted about 60 to 90 minutes. Data collection took place across a two-month period (March–April 2026). | Pages 12–13 |
| **22** | Data saturation | Was data saturation discussed? | Yes. Saturation informed recruitment: interviews were transcribed and coded during fieldwork, and fieldwork continued until new interviews produced the same information as earlier ones. It was a narrative judgement; quantitative saturation metrics were not used. | Pages 11–12 |
| **23** | Returning transcripts | Were transcripts returned to participants for comment and/or correction (member checking)? | Not reported. | N/A |
| **DOMAIN 3: Data analysis and findings** | | | | |
| ***Data analysis*** | | | | |
| **24** | Number of data coders | How many data coders were used? | One coder. Formal coding and theme development were conducted by the corresponding author, with review of the coding log and memo trail by two senior co-authors. 66 codes, 20 sub-themes and 6 final themes were generated (Table 2). | Pages 8, 15; Table 2 on page 17 |
| **25** | Description of the coding tree | Did authors provide a description of the coding tree? | Yes. Table 2 (p. 17) presents all six themes, their sub-themes, and corresponding meaningful units (codes), forming a complete coding structure. | Page 17; Table 2 on page 17 |
| **26** | Derivation of themes | Were themes identified in advance or derived from the data? | Themes were derived inductively. Thematic analysis moved iteratively between participants' language and broader interpretive concepts. The SEM was applied subsequently as an organising framework rather than a deductive coding template. | Pages 13–14 |
| **27** | Software | What software, if applicable, was used to manage the data? | No qualitative data management software is reported. Analysis was conducted through direct iterative engagement with transcripts. | Page 13 |
| **28** | Participant checking | Did participants provide feedback on the findings? | Not reported. | N/A |
| ***Reporting*** | | | | |
| **29** | Quotations presented | Were participant quotations presented to illustrate the themes/findings? | Yes. Verbatim quotations presented throughout the Results section for all six themes and their sub-themes, in italics, attributed by participant code (e.g., IDI_16). | Pages 18–26 |
| **30** | Data and findings consistent | Was there consistency between the data presented and the findings? | Yes. Each theme and sub-theme is directly supported by verbatim quotations. Table 2 maps codes to sub-themes and themes. Divergent accounts (e.g., co-existence of positive survival evaluation with reports of pain, privacy violations, and financial burden) are explicitly acknowledged. | Pages 17–26 |
| **31** | Clarity of major themes | Were major themes clearly presented in the findings? | Yes. Six clearly labelled major themes, each with a dedicated Results sub-section, descriptive narrative, and supporting quotations. Table 2 provides a full structured overview of all themes, sub-themes, and codes. | Pages 17–26; Table 2 on page 17 |
| **32** | Clarity of minor themes | Is there a description of diverse cases or discussion of minor themes? | Yes. Divergent experiences are foregrounded throughout Results and Discussion — including contrasting accounts of positive and negative communication, presence vs. absence of social support, and post-discharge uncertainty. Minor themes such as self-blame, reproductive anxiety, and selective social disclosure are discussed. | Pages 24–26, 30–34 |

Note N/A- not applicable for study design
