# Supplementary material for: Women’s experiences of emergency post-abortion care at Kawempe National Referral Hospital, Uganda: A qualitative descriptive study
Source: PLoS One. 2026 Nov 5;21(11):e0357615. doi: 10.1371/journal.pone.0357615 (PMC13739178; doi:10.1371/journal.pone.0357615)
Supplement: Supplementary file 4 [file pone.0357615.s004.docx]

**Response Letter to Reviewers**

**Manuscript PONE-D-26-31923**

*Women's experiences of emergency post-abortion care at Kawempe National Referral Hospital, Uganda: a qualitative descriptive study*

Dear Academic Editor and Reviewers,

We thank the Academic Editor and both reviewers for their careful and constructive engagement with our manuscript. We have revised the manuscript substantially in response to every point raised. Below we respond to each comment in turn, indicating the corresponding change in the manuscript. Line numbers refer to the clean revised manuscript submitted alongside this letter.

# A. Journal Requirements

## 1. PLOS ONE style requirements, including file naming

***Comment:*** *Please ensure that your manuscript meets PLOS ONE's style requirements, including those for file naming.*

**Response:** We have reviewed the manuscript against the PLOS ONE Manuscript Body Formatting Guidelines and the Title/Author/Affiliations Formatting Guidelines. Heading levels, sentence-case headings, figure/table placement conventions, and reference formatting (numbered citation-sequence, Vancouver/ICMJE style) have been checked throughout. Files will be named per PLOS ONE convention at upload (Manuscript, Response to Reviewers, Revised Manuscript with Track Changes).

## 2. Data Availability Statement contact point

***Comment:*** *In the online submission form you indicate that your data is not available for proprietary reasons and have provided a contact point for accessing this data. Please note that your current contact point is a co-author on this manuscript. According to our Data Policy, the contact point must not be an author on the manuscript and must be an institutional contact, ideally not an individual. Please revise your data statement to a non-author institutional point of contact...*

**Response:** The Data Availability Statement has been revised. The contact point is now Makerere University College of Health Sciences, Department of Obstetrics and Gynaecology, P.O. Box 7072, Kampala, Uganda (email: dean@chs.mak.ac.ug), which is an institutional contact and not a manuscript author. Requests remain subject to approval from SOMREC (reference Mak-SOMREC-2025-913). See Data availability statement, and the corresponding online submission form field, which has been updated to match.

## 3. Ethics statement location

***Comment:*** *Your ethics statement should only appear in the Methods section of your manuscript.*

**Response:** Confirmed. The ethics statement appears only in Methods > Ethics statement. We have checked the Abstract, Results, Discussion, and Conclusions and confirmed no duplicate or partial ethics statement appears elsewhere.

## 4. Reviewer-suggested citations

***Comment:*** *If the reviewer comments include a recommendation to cite specific previously published works, please review and evaluate these publications to determine whether they are relevant and should be cited. There is no requirement to cite these works unless the editor has indicated otherwise.*

**Response:** We reviewed both works suggested by Reviewer 2 and judged them relevant to strengthening the legal/bioethical framing of our Introduction. Both have been cited: Cioffi et al. (2023, Healthcare) and Ishola et al. (2021, Health Policy and Planning), now references 13 and 14. See Introduction, paragraph beginning “Uganda’s legal restriction of abortion is often described narrowly...”

# B. Reviewer 1

***Comment:*** *Women's experience in EPAC is very crucially related to the type of abortion undergone, possible complication undergone and the treatment options provided.*

**Response:** We agree, and Table 1 has been expanded to report, for all sixteen participants: gestational age at presentation, presenting complication, treatment received, transfusion need, and referral status from another facility. Whether presentation followed miscarriage or induced abortion was not systematically captured in the clinical or interview records available for this analysis; this is now stated explicitly in Study population and data source, and its interpretive consequences (bounding stigma/guilt interpretations to narratives where this context was disclosed) are addressed in Strengths and limitations.

***Comment:*** *There are six broad areas mentioned as outline for the women's experience, but the data mentioned does not reflect some of the areas (e.g., the referral system is mentioned of other health facilities where the women has encountered and not the experience of the referral from the study site).*

**Response:** This is a fair characterisation of the data's scope, and we have made that scope explicit rather than implicit. The interview guide (S1 File) contained no dedicated question on referral or transfer at any stage; pre-arrival referral content emerged through the open-ended opening question and its “step by step” probe, while referral or transfer between departments within KNRH once admitted (e.g., reception, the gynaecological emergency unit, and theatre) was neither asked about nor volunteered. We have revised the opening of Theme 5 (Structural barriers across the pathway of care) to state this directly, and added a corresponding point to Strengths and limitations noting this as a direction for future work — adding a dedicated question on intra-facility transfer specifically.

***Comment:*** *It also would have been good to see the PAC pathway experience by the level of providers as there are different cadres of providers providing the services in the study site.*

**Response:** We agree this would strengthen the analysis, but provider cadre (e.g., consultant, resident, intern, midwife, nursing officer) was not systematically recorded during data collection; participants distinguish providers only descriptively (e.g., “the doctor”, “a female doctor”). We have added this as an explicit limitation in Strengths and limitations, stating plainly that this analysis cannot establish whether experiences of pain management, communication, or dignity differed systematically by provider cadre, as opposed to by individual provider or by moment in the care pathway, and flagging this as a design consideration for future EPAC research at KNRH.

# C. Reviewer 2

We thank Reviewer 2 for an exceptionally thorough review. We address each point below in the order raised.

***Comment:*** *The designation of the study as phenomenological is not sufficiently substantiated, as the authors describe an inductive thematic analysis without specifying the phenomenological orientation or the analytical method actually employed.*

**Response:** We agree and have removed the phenomenological designation throughout (title, abstract, Methods). The study is now described as a qualitative descriptive design using inductive thematic analysis, with an explicit statement that no specific phenomenological, grounded-theory, or narrative analytic tradition was adopted. See Study design.

***Comment:*** *The undifferentiated use of the term “post-abortion care”... The authors should therefore clarify whether the sample included women following miscarriage, induced abortion, or both; whether this information was collected; and how it was considered in the analysis.*

**Response:** Clarified: this distinction was not systematically recorded in the clinical intake or interview protocol and was not used as an inclusion or exclusion criterion. We now state this directly in Study population and data source, and we have bounded our interpretation accordingly: stigma, guilt, and moral-vulnerability findings (Themes 2 and 4) are presented as grounded in the subset of narratives where participants themselves disclosed or implied the nature of the pregnancy loss, not as characterising the sample as a whole. This constraint is discussed further in Strengths and limitations.

***Comment:*** *The description of the Ugandan legal context should be expanded to consider not only the formal restrictiveness of abortion legislation but also its potential implications for the right to health, self-determination, and timely access to care... [suggested] https://doi.org/10.3390/healthcare11040520 and 10.1093/heapol/czab069.*

**Response:** Expanded. A new paragraph in the Introduction (immediately following the existing legal/stigma paragraph) discusses restrictive abortion law as a determinant of health inequity and self-determination, citing Cioffi et al. (2023) for the bioethical/rights framing and Ishola et al. (2021) for empirical evidence that abortion law liberalisation is associated with improved health-service access and outcomes in LMICs, with restrictive regimes linked to delayed care-seeking and unsafe methods. Both are now references 13 and 14.

***Comment:*** *The statement that “approximately half of all abortions worldwide occur under unsafe conditions” should report the exact proportion provided by the cited source, the period to which the estimate refers, and the definition of “unsafe abortion” adopted... Rhetorical expressions such as “often the difference between life and death” should be replaced with more measured wording.*

**Response:** Both corrected. The statistic now reads: “an estimated 45.1% of the 55.7 million abortions performed annually between 2010 and 2014 were unsafe, defined by WHO as a procedure to terminate a pregnancy carried out either by a person lacking the necessary skills or in an environment not meeting minimal medical standards, or both.” The “life and death” phrase has been replaced with: “timely access is strongly associated with reduced maternal morbidity and mortality.” See Introduction, paragraph 2.

***Comment:*** *The knowledge gap should be more clearly delineated... The existing studies should be briefly and critically synthesised, and the authors should specify which dimension remains insufficiently explored.*

**Response:** A new paragraph has been added synthesising the existing Ugandan literature by what each strand actually covers (utilisation-focused quantitative studies vs. the one comparable qualitative satisfaction study) and stating explicitly what none of it addresses: EPAC specifically as a time-critical emergency presentation, and how women themselves weigh survival, dignity, and structural constraint against one another. See Introduction, paragraph immediately before “This study, therefore, explored...”.

***Comment:*** *The role of the Socio-Ecological Model (SEM) should also be described consistently... The authors should clarify whether the model informed the interview guide and coding process or was applied only after theme development.*

**Response:** Clarified throughout. Study design and Data analysis now state explicitly that the SEM was applied post hoc, as an organising interpretive lens over inductively derived themes, not as an a priori framework shaping the interview guide or coding. The Introduction's closing paragraph and the Discussion's SEM subsection have been revised to match this framing consistently, and the Discussion now explicitly distinguishes conclusions grounded directly in participants' accounts from those introduced through the SEM's organising structure.

***Comment:*** *If the authors intend to retain the phenomenological designation, they should specify the epistemological orientation... Otherwise, the study may be more accurately described as an exploratory qualitative study using thematic analysis. The EPAC population should also be clinically defined.*

**Response:** As above, the study is now described as qualitative descriptive with inductive thematic analysis. The EPAC population is now clinically characterised in the expanded Table 1 (gestational age, presenting complication, treatment received, transfusion need, referral status). We chose qualitative description over the reviewer's suggested alternative of "exploratory qualitative study" because "exploratory" describes a research purpose rather than a named analytic tradition, and does not specify how the data were analysed or what evidentiary standard the findings should be held to. Qualitative description, following Sandelowski (2000, 2010), is defined by staying close to the data with minimal researcher inference and producing a comprehensive, low-interpretation account in participants' own language, without committing to a specific theoretical lens; this matches both our analytic approach and the six fully-developed, sub-themed findings reported, which extend beyond a preliminary or scoping account.

***Comment:*** *The sampling strategy requires greater transparency... The authors should therefore explain how the final sample of 16 participants was reached and describe the operational criteria used to determine data saturation.*

**Response:** Sampling strategy has been expanded: we now report that 24 women meeting eligibility criteria were approached, of whom 16 consented and 8 declined, and specify that maximal variation was operationalised using the characteristics summarised in Table 1. The saturation paragraph now states explicitly that saturation was assessed narratively (consistent with, but not applying, Guest, Namey and Chen's quantitative tracking metrics), and that this is a qualitative judgement rather than a metrics-based determination.

***Comment:*** *Recruitment also appears to have occurred in connection with a scheduled review appointment, whereas some participants reported receiving no follow-up. This apparent inconsistency should be resolved.*

**Response:** Resolved. Data collection now states explicitly that recruitment deliberately targeted the scheduled post-discharge review appointment (occurring around two weeks after discharge under standard clinical practice at the unit) rather than the point of discharge itself, and that this is consistent with the interval-to-interview data now reported. We have also added a limitation noting that participants interviewed at this point had already reached, or nearly reached, the standard follow-up window, which should be read alongside Theme 6's account of perceived follow-up absence and the kind of follow up that the patient was looking for was perhaps a daily follow-up or after 2 days or at home and not necessarily a follow up review date which she saw as mandatory.

***Comment:*** *The manuscript also lacks an adequate account of researcher reflexivity. The authors should briefly describe the researchers' professional roles, their relationships with the institution and participants, and the strategies used to recognise and limit the influence of their assumptions.*

**Response:** A new subsection, Researcher positionality, has been added to Methods. It describes all authors' clinical affiliation with KNRH/Makerere; that interviews were conducted by the corresponding author (a postgraduate trainee in the same department) with research assistants who were registered midwives employed at KNRH facilitating participant approach and consent; that this insider position extended to the interview encounter itself, not only to analysis; the single-analyst coding process and its senior-author review; and the reflexive journaling and bracketing procedure applied at three points (a pre-data-collection session recording assumptions and clinical background, individual post-interview reflexive journals kept separately by each interviewer, and analytic memos reviewed in team coding meetings), used specifically to check clinically-derived codes against participants' own words.

***Comment:*** *Table 1 should also report... the principal clinical characteristics: miscarriage or induced abortion, where known; gestational age; presenting complication; treatment received; need for transfusion; referral from another facility; and interval between discharge and interview.*

**Response:** Table 1 has been expanded with all of these variables except miscarriage/induced abortion status, which was not captured (explained above and in Study population and data source). The added rows report, for all sixteen participants: gestational age at presentation, presenting complication, treatment received, transfusion need, referral status from another facility, and interval between discharge and interview.

***Comment:*** *Statements regarding costs also require clarification... The quotation referring to money required in connection with a blood transfusion does not appear fully consistent with the description of EPAC as free of charge.*

**Response:** Clarified. A new passage in Theme 5 distinguishes formal facility charges (ultrasound scans, medications out of stock at the pharmacy, consistent with EPAC itself being officially free) from informal payment: in the account concerning blood transfusion, money was given directly to health workers not as a billed fee for the blood product or its screening, but as an informal incentive intended to motivate staff to act quickly in sourcing blood for an urgent transfusion. We note this is consistent with informal payment practices documented elsewhere in under-resourced public health systems.

***Comment:*** *Particular attention should also be paid to the participant reporting symptoms potentially suggestive of infection. The authors should clarify whether the research protocol included clinical assessment or referral procedures for health concerns identified during an interview.*

**Response:** Clarified in Theme 6: the study protocol included a defined procedure for referring participants for clinical assessment where concerning symptoms were disclosed mid-interview; no case during data collection required this procedure to be invoked.

***Comment:*** *The Discussion offers an interesting interpretation... but several conclusions extend beyond what the data can support... Expressions such as “fear-driven reordering”, “coercive”, “produced”, and “the SEM resolved” should therefore be replaced with more cautious wording.*

**Response:** All four phrases have been revised. “Fear-driven reordering” is replaced with a direct description of acute mortal threat leading women to prioritise survival, temporarily subordinating relational and procedural quality dimensions. The passage previously implying coercion now describes judgmental communication as “compounded disrespect” given that women in acute emergency presentations cannot exit the care relationship. “The SEM resolved” is replaced with “The SEM provided a coherent multi-level account of the conditions under which clinical success and relational failure simultaneously occurred.” We reviewed the Discussion throughout for similarly unsupported causal language.

***Comment:*** *The comparison with the literature should clarify which findings are genuinely novel rather than stating generically that the study confirms and extends previous evidence.*

**Response:** The Discussion's engagement with prior literature (Baum, Ouedraogo, Cleeve, Lythgoe, Udho, Stillman, Kabunga, and others) has been reviewed to ensure each comparison states specifically what this study confirms, extends, or adds, rather than a generic confirms-and-extends framing; see Discussion, paragraphs 2–6.

***Comment:*** *The Limitations section should more fully acknowledge potential selection and social desirability biases, the influence of the setting and researchers, translation-related issues, the clinical heterogeneity of the sample, and the absence of corroborating clinical or organisational data.*

**Response:** Strengths and limitations has been substantially expanded to address: the two-stage recruitment process potentially under-sampling women most fearful of re-engaging with the health system; the 33% refusal rate (8 of 24) as an uncharacterised source of possible selection bias; institutional social-desirability pressure arising from interviewing on hospital premises during a dependent clinical relationship; a power differential between the research team and a participant group of whom 62.5% had at most lower secondary education; the timing confound between recruitment window and standard follow-up scheduling; unrecorded geographic origin and religious affiliation; unrecorded uterine evacuation procedure type; the miscarriage/induced abortion distinction; translation conducted by the interviewers themselves rather than independent translators; and the intra-facility referral and provider-cadre gaps raised by Reviewer 1.

***Comment:*** *The Conclusions adequately reflect the central message of the study but should be explicitly confined to the experiences of the 16 participants. The assertion that post-discharge recovery was largely unstructured should be tempered, while stigma should not be characterised as a “direct determinant” in the absence of causal evidence.*

**Response:** Conclusions have been revised: the opening sentence now reads “Among the sixteen women interviewed...” and explicitly scopes the survival-is-necessary-but-not-sufficient claim to this sample. The recovery statement now reads “recovery experienced by these participants as largely unstructured” rather than a general claim. “Stigma as a direct determinant of care quality” has been replaced with language noting that stigma shaped how care was communicated and experienced, while its causal contribution to objective care quality was not directly tested.

# **Reference cited in this letter**

Sandelowski, M., 2000. Whatever happened to qualitative description? Research in Nursing & Health, 23(4), pp.334–340.

Sandelowski, M., 2010. What's in a name? Qualitative description revisited. Research in Nursing & Health, 33(1), pp.77–84.

# Closing

We believe these revisions have substantially strengthened the manuscript's methodological transparency, the precision of its claims, and its engagement with the existing literature and legal context. We thank the Academic Editor and both reviewers again for their time and constructive guidance, and hope the revised manuscript now meets PLOS ONE's criteria for publication.

On behalf of all authors,

Kawungu Saad Sessimba (corresponding author)
