# Supplementary material for: Women’s experiences of emergency post-abortion care at Kawempe National Referral Hospital, Uganda: A qualitative descriptive study
Source: PLoS One. 2026 Nov 5;21(11):e0357615. doi: 10.1371/journal.pone.0357615 (PMC13739178; doi:10.1371/journal.pone.0357615)
Supplement: Supplementary file 5 [file pone.0357615.s005.docx]

**Response Letter to Reviewers**

**Manuscript PONE-D-26-31923R1**

*Women's experiences of emergency post-abortion care at Kawempe National Referral Hospital, Uganda: a qualitative descriptive study*

Dear Academic Editor and Reviewers,

We thank the Academic Editor and Reviewer 2 for their careful second-round review and comments plus guidance. Reviewer 2 raised six points, and the journal asked us to check the reference list. We respond to each in turn below, indicating the corresponding change in the manuscript. Line numbers refer to the clean revised manuscript submitted alongside this letter.

A. Journal Requirements

1. Reviewer-suggested citations

***Comment:*** *If the reviewer comments include a recommendation to cite specific previously published works, please review and evaluate these publications to determine whether they are relevant and should be cited. There is no requirement to cite these works unless the editor has indicated otherwise.*

**Response:** Reviewers did not recommend specific publications in this round, so we have added no citations on that basis.

2. Reference list

***Comment:*** *Please review your reference list to ensure that it is complete and correct. If you have cited papers that have been retracted, please include the rationale for doing so in the manuscript text, or remove these references and replace them with relevant current references. Any changes to the reference list should be mentioned in the rebuttal letter that accompanies your revised manuscript...*

**Response:** We checked all 37 references for completeness and accuracy. No reference was added, removed or replaced. We also screened all 37 references against the Retraction Watch database and found no retracted articles.

B. Reviewer 2

Statistical analysis

***Comment:*** *Has the statistical analysis been performed appropriately and rigorously? Reviewer 2: "I Don't Know".*

**Response:** This is a qualitative study, and no statistical tests were run. Table 1 reports participant counts only.

1. Table 1: interval between discharge and interview

***Comment:*** *"the Authors state in their response letter that Table 1 has been expanded to include the interval between discharge and interview, but this information does not appear in the revised table."*

**Response:** Our first response listed the interval among the rows added to Table 1, and the row was not in the submitted table. After discussion we removed it, because the interval is the same for every participant, and we did not update the letter. We should have. Every participant was interviewed at least 14 days after discharge, and the number of days beyond 14 was not recorded for individual participants, so a row reading "14 days or more" for 16 of 16 would add nothing the text does not already say. We have therefore left Table 1 as it was and state the point where it belongs, in the text. The criterion is set out in Sampling strategy (lines 214–216), and Data collection (lines 246–247) now says that all sixteen participants were interviewed at least 14 days after discharge and that the exact number of days beyond 14 was not recorded. We also deleted a clause in Data collection that sent the reader to Table 1 for this interval.

2. Table 1 and the limitations section: treatment received

***Comment:*** *"Table 1 specifies the treatment received by all sixteen participants (manual vacuum aspiration, misoprostol-only management, and sharp curettage), whereas the Strengths and limitations section (lines 642–644) states that the type of uterine evacuation procedure was not systematically recorded."*

**Response:** The two statements contradicted each other. Table 1 reports the procedure for all 16 participants, so we deleted the sentence saying it was not systematically recorded. The limitation that remains is narrower and is stated in its place (lines 655–659): procedure type was not used as an analytic variable, and with 16 participants the procedural pain findings in Theme 2 cannot be compared across procedures or attributed to a particular one.

3. Theme 5: money paid in connection with blood transfusion

***Comment:*** *"Theme 5 describes the money associated with a blood transfusion as an informal payment made directly to healthcare workers to expedite care. However, the participant's account quoted in the manuscript does not seem to support this specific interpretation."*

**Response:** We agree that the quotation alone does not show where the money went. The explanation now sits directly under the IDI_2 quotation in Theme 3 (lines 390–397), where it belongs, and not in Theme 5. It sets out the setting: under hospital policy EPAC at KNRH is free of charge except for radiological services, which all patients pay for out of pocket, so no fee should have been billed for the transfusion. Study setting (lines 189–191) now states this policy. The text says that IDI_2 did not say who received the money or what it was for, and adds a statement from the same interview: "...and the little money we gave here and there helped us get things done faster." Read together, the two statements lead us to interpret the money as an attempt to speed up care and not as payment for blood or tests. We also removed two statements we could not support from the interviews: that this matched informal payment practices documented elsewhere, and that women found the line between free care and ancillary costs distressing. The Theme 5 paragraph (lines 445–449) is now a short pointer back to Theme 3.

4. IDI_6: symptoms disclosed during the interview

***Comment:*** *"I would also appreciate some clarification regarding participant IDI_6, who reported persistent lower abdominal pain and what she described as potentially purulent discharge during the interview (lines 471–478)... it remains unclear why they were not considered sufficient to warrant further clinical assessment."*

**Response:** We agree that these symptoms do not establish infection but would ordinarily prompt a clinical assessment. Our earlier statement that no case required the referral procedure did not explain why her account was not treated as one, so we deleted it from Theme 6 and replaced the sentence in Strengths and limitations with a pointer to Theme 6 (lines 475–482 and 662–664). We also restored her full quotation (lines 470–474), which had been abridged and left out her words "I think something is wrong". Theme 6 now says that IDI_6 was interviewed at her scheduled review appointment at the gynaecological emergency unit, that the protocol provided for referral of any participant who disclosed concerning symptoms during an interview, and that she was therefore already in the unit for review and care. It also says that she agreed to continue the interview because her symptoms were not too severe for her, and that, with her consent, the reviewing clinician was made aware of the symptoms she had disclosed.

5. Data saturation in a secondary analysis

***Comment:*** *"Since the manuscript now identifies the study as a secondary thematic analysis of interviews collected within a broader study, it would be useful to specify whether saturation informed the original recruitment process or was assessed retrospectively."*

**Response:** Saturation did inform recruitment, and our previous wording did not make that clear. Data collection and analysis ran side by side. Interviews were transcribed and coded while fieldwork was still under way, and themes were generated as more women were interviewed. Fieldwork continued until new interviews produced the same information as earlier ones, and no interviews were conducted after that point. Sampling strategy now says this (lines 227–238). It also says that the judgement is narrative and did not use quantitative metrics.

6. Absence of proactive contact versus absence of scheduled follow-up

***Comment:*** *"I would also suggest distinguishing more clearly between the absence of proactive contact after discharge and the absence of scheduled follow-up appointments, as these are not necessarily equivalent and may have different implications for the interpretation of the findings."*

**Response:** We have made this distinction in four places. Theme 6 (lines 467–469 and 475–490) now reports that participants described no contact from the hospital after discharge, such as a telephone call. It notes that every participant was interviewed at a scheduled review appointment, so a review visit existed for the women in this sample, and it sets out the different implications: a missing call points to a gap in outreach, while a missing appointment would point to a gap in access to review. Our data speak to the first only. In Table 2 (page 17) the sub-theme is renamed "No proactive contact and continuity gaps" and the code "No review" is replaced, since it conflicted with the fact that participants attended a review. The Discussion (line 570) now says "no system for contacting women after discharge" in place of "absent follow-up systems". Strengths and limitations (lines 635–637) adds that all participants were recruited at a review appointment, so women who were never offered one, or did not attend, are absent from the data.

C. Supporting file

S2 File (COREQ) has been updated so that its page numbers match the revised clean manuscript. We also corrected its responses on saturation, coding, field notes and sample characteristics to match the revised text, and its author list to match the title page. COREQ item 21 now gives the interview duration (about 60 to 90 minutes), which Data collection also reports (lines 258–259).

Closing

We believe these revisions have resolved the remaining points raised and have made the manuscript clearer and more consistent. We thank the Academic Editor and Reviewers again for their time and careful guidance, and hope the revised manuscript now meets PLOS ONE's criteria for publication.

On behalf of all authors,

Kawungu Saad Sessimba (corresponding author)
