# Supplementary material for: Women’s experiences of emergency post-abortion care at Kawempe National Referral Hospital, Uganda: A qualitative descriptive study
Source: PLoS One. 2026 Nov 5;21(11):e0357615. doi: 10.1371/journal.pone.0357615 (PMC13739178; doi:10.1371/journal.pone.0357615)
Supplement: Peer review history

# Peer review: Decision Letter 0

**DOI:** 10.1371/journal.pone.0357615.r001

**Authors:** Andrea Cioffi

20 Sep 2026

Dear Dr. Saad Sessimba,

Thank you for submitting your manuscript to PLOS One. After careful consideration, we feel that it has merit but does not fully meet PLOS One’s publication criteria as it currently stands. Therefore, we invite you to submit a revised version of the manuscript that addresses the points raised during the review process.

Please submit your revised manuscript by Nov 19 2026 11:59PM. If you will need more time than this to complete your revisions, please reply to this message or contact the journal office at plosone@plos.org. When you're ready to submit your revision, log on to https://www.editorialmanager.com/pone/ and select the 'Submissions Needing Revision' folder to locate your manuscript file.

- A letter that responds to each point raised by the academic editor and reviewer(s). You should upload this letter as a separate file labeled 'Response to Reviewers'.
- A marked-up copy of your manuscript that highlights changes made to the original version. You should upload this as a separate file labeled 'Revised Manuscript with Track Changes'.
- An unmarked version of your revised paper without tracked changes. You should upload this as a separate file labeled 'Manuscript'.

If you would like to make changes to your financial disclosure, please include your updated statement in your cover letter. Guidelines for resubmitting your figure files are available below the reviewer comments at the end of this letter.

If applicable, we recommend that you deposit your laboratory protocols in protocols.io to enhance the reproducibility of your results. Protocols.io assigns your protocol its own identifier (DOI) so that it can be cited independently in the future. For instructions see: https://journals.plos.org/plosone/s/submission-guidelines#loc-laboratory-protocols. Additionally, PLOS One offers an option for publishing peer-reviewed Lab Protocol articles, which describe protocols hosted on protocols.io. Read more information on sharing protocols at https://plos.org/protocols?utm_medium=editorial-email&utm_source=authorletters&utm_campaign=protocols.

As the corresponding author, your ORCID iD is verified in the submission system and will appear in the published article. PLOS supports the use of ORCID, and we encourage all coauthors to register for an ORCID iD and use it as well. Please encourage your coauthors to verify their ORCID iD within the submission system before final acceptance, as unverified ORCID iDs will not appear in the published article. *Only*  the individual author can complete the verification step; PLOS staff *cannot*  verify ORCID iDs on behalf of authors.

We look forward to receiving your revised manuscript.

Kind regards,

Andrea Cioffi

Academic Editor

PLOS One

Journal Requirements:

When submitting your revision, we need you to address these additional requirements.

1. Please ensure that your manuscript meets PLOS ONE’s style requirements, including those for file naming. The PLOS ONE style templates can be found at

https://journals.plos.org/plosone/s/file?id=wjVg/PLOSOne_formatting_sample_main_body.pdf and

https://journals.plos.org/plosone/s/file?id=ba62/PLOSOne_formatting_sample_title_authors_affiliations.pdf.

2. In the online submission form you indicate that your data is not available for proprietary reasons and have provided a contact point for accessing this data. Please note that your current contact point is a co-author on this manuscript. According to our Data Policy, the contact point must not be an author on the manuscript and must be an institutional contact, ideally not an individual. Please revise your data statement to a non-author institutional point of contact, such as a data access or ethics committee, and send this to us via return email. Please also include contact information for the third party organization, and please include the full citation of where the data can be found.

3. Your ethics statement should only appear in the Methods section of your manuscript. If your ethics statement is written in any section besides the Methods, please delete it from any other section.

4. If the reviewer comments include a recommendation to cite specific previously published works, please review and evaluate these publications to determine whether they are relevant and should be cited. There is no requirement to cite these works unless the editor has indicated otherwise.

[Note: HTML markup is below. Please do not edit.]

Reviewers' comments:

Reviewer’s Responses to Questions

**Comments to the Author**

1. Is the manuscript technically sound, and do the data support the conclusions?

Reviewer #1: Partly

Reviewer #2: Partly

**********

2. Has the statistical analysis been performed appropriately and rigorously?

Reviewer #1: Yes

Reviewer #2: I Don't Know

**********

3. Have the authors made all data underlying the findings in their manuscript fully available?

The PLOS Data policy

Reviewer #1: No

Reviewer #2: Yes

**********

4. Is the manuscript presented in an intelligible fashion and written in standard English?

Reviewer #1: Yes

Reviewer #2: Yes

**********

Reviewer #1: Women’s experience in EPAC is very crucially related to the type of abortion undergone, possible complication undergone and the treatment options provided. There are six broad areas mentioned as outline for the women’s experience, but the data mentioned does not reflect some of the areas (e.g., the referral system is mentioned of other health facilities where the women has encountered and not the experience of the referral from the study site).

The document is attached for the authors to address to make the article more robust. It also would have been good to see the PAC pathway experience by the level of providers as there are different cadres of providers providing the services in the study site.

Reviewer #2: First, I would like to thank the Editor for the opportunity to review this manuscript.

The authors address a relevant topic of considerable public health importance. The manuscript provides a valuable perspective on women’s experiences of emergency post-abortion care (EPAC); however, several conceptual, methodological, and interpretative issues require substantial clarification to strengthen its scientific rigour.

The abstract clearly presents the study objective and main findings, but the methodological description should be more precise. In particular, the designation of the study as phenomenological is not sufficiently substantiated, as the authors describe an inductive thematic analysis without specifying the phenomenological orientation or the analytical method actually employed.

An important concern relates to the undifferentiated use of the term “post-abortion care”, which may encompass complications following both miscarriage and induced abortion, including potentially unsafe abortion. Although these conditions may present similarly during the emergency phase, they can entail profoundly different emotional, social, and legal experiences. In a study focused on lived experience, grief following pregnancy loss, stigma, guilt, fear of judgement, and concern about legal consequences cannot be assumed to apply uniformly to all participants. The authors should therefore clarify whether the sample included women following miscarriage, induced abortion, or both; whether this information was collected; and how it was considered in the analysis. If this distinction was unavailable, interpretations concerning stigma and moral vulnerability should be limited to the narratives in which these dimensions explicitly emerged, and this uncertainty should be acknowledged as a study limitation.

The description of the Ugandan legal context should be expanded to consider not only the formal restrictiveness of abortion legislation but also its potential implications for the right to health, self-determination, and timely access to care. To this end, the authors may wish to integrate both medico-legal and bioethical contributions, such as the article identified by https://doi.org/10.3390/healthcare11040520 , and empirical evidence on the effects of abortion legislation in low- and middle-income countries, such as the systematic review 10.1093/heapol/czab069. These references could strengthen the link between legal restrictions, inequalities in access to care, and health outcomes.

The epidemiological discussion of unsafe abortion also requires greater precision. The statement that “approximately half of all abortions worldwide occur under unsafe conditions” should report the exact proportion provided by the cited source, the period to which the estimate refers, and the definition of “unsafe abortion” adopted. Moreover, unsafe abortion and EPAC should not be presented as interchangeable concepts, since miscarriage may also result in haemorrhage, sepsis, or retained products of conception. Rhetorical expressions such as “often the difference between life and death” should be replaced with more measured wording referring to reductions in maternal morbidity and mortality.

The knowledge gap should be more clearly delineated. The general statement that qualitative evidence from Uganda is limited is insufficient to establish the originality of the study, particularly because the authors cite previous research on women’s experiences of post-abortion care. The existing studies should be briefly and critically synthesised, and the authors should specify which dimension remains insufficiently explored—for example, the emergency nature of care, the national referral hospital setting, the post-discharge period, or the multilevel interpretation of women’s experiences.

The role of the Socio-Ecological Model (SEM) should also be described consistently. The Introduction suggests that the model guided the examination of individual, interpersonal, facility, and community factors from the outset, whereas the Methods state that themes were generated inductively and only subsequently organised through the SEM. The authors should clarify whether the model informed the interview guide and coding process or was applied only after theme development. In the latter case, it should be presented as a subsequent interpretative lens. The authors should also explain what specific analytical value the SEM adds beyond simply classifying findings across different levels.

The Methods section requires substantial expansion. If the authors intend to retain the phenomenological designation, they should specify the epistemological orientation, analytical method, and procedures consistent with that approach. Otherwise, the study may be more accurately described as an exploratory qualitative study using thematic analysis. The EPAC population should also be clinically defined, including, where available, the circumstances of pregnancy termination, the presenting complications, and the treatments received.

The sampling strategy requires greater transparency. The authors report using purposive maximum-variation sampling but do not specify which characteristics were considered a priori when selecting participants. The presence of women differing in age or educational level in the final sample does not, in itself, demonstrate that these variables guided recruitment. The authors should therefore explain how the final sample of 16 participants was reached and describe the operational criteria used to determine data saturation.

Recruitment also appears to have occurred in connection with a scheduled review appointment, whereas some participants reported receiving no follow-up. This apparent inconsistency should be resolved. The authors should also clarify whether women who did not return to the hospital were excluded, as such a recruitment strategy may have omitted those experiencing the greatest barriers to access or the most negative care experiences.

The manuscript also lacks an adequate account of researcher reflexivity. The authors should briefly describe the researchers’ professional roles, their relationships with the institution and participants, and the strategies used to recognise and limit the influence of their assumptions on data collection and interpretation. This is particularly important in a study addressing abortion, stigma, and participants’ evaluations of care received within the same institution.

Regarding the Results, participant descriptions are limited to sociodemographic characteristics. Table 1 should also report, in aggregate form and in a manner compatible with confidentiality, the principal clinical characteristics: miscarriage or induced abortion, where known; gestational age; presenting complication; treatment received; need for transfusion; referral from another facility; and interval between discharge and interview. These variables may substantially influence pain, fear, perceptions of care, and subsequent concerns.

Statements regarding costs also require clarification. The authors should distinguish between formal charges, external purchases of medicines or supplies, transportation expenses, and other indirect costs. The quotation referring to money required in connection with a blood transfusion does not appear fully consistent with the description of EPAC as free of charge. Particular attention should also be paid to the participant reporting symptoms potentially suggestive of infection. The authors should clarify whether the research protocol included clinical assessment or referral procedures for health concerns identified during an interview.

The Discussion offers an interesting interpretation of the coexistence of clinical improvement and negative care experiences, but several conclusions extend beyond what the data can support. The authors should consistently distinguish participants’ perceptions from objective assessments of clinical and organisational quality. The study documents severe pain but cannot establish inadequate analgesia without information on the treatments administered. It describes post-discharge uncertainty but does not demonstrate the absence of a formal follow-up system. It also reports perceived structural difficulties but cannot determine how these factors causally influenced provider behaviour. Expressions such as “fear-driven reordering”, “coercive”, “produced”, and “the SEM resolved” should therefore be replaced with more cautious wording.

The comparison with the literature should clarify which findings are genuinely novel rather than stating generically that the study confirms and extends previous evidence. The Limitations section should more fully acknowledge potential selection and social desirability biases, the influence of the setting and researchers, translation-related issues, the clinical heterogeneity of the sample, and the absence of corroborating clinical or organisational data. The presence of negative accounts is not sufficient to exclude social desirability bias.

The Conclusions adequately reflect the central message of the study but should be explicitly confined to the experiences of the 16 participants. The assertion that post-discharge recovery was largely unstructured should be tempered, while stigma should not be characterised as a “direct determinant” in the absence of causal evidence. More broadly, implications directly supported by participants’ accounts—such as respectful communication, attention to pain, privacy, and clear discharge information—should be distinguished from organisational recommendations concerning triage procedures, resource availability, and referral-system redesign, which would require additional evidence.

**********

what does this mean?). If published, this will include your full peer review and any attached files.

If you choose “no”, your identity will remain anonymous but your review may still be made public.

**Do you want your identity to be public for this peer review?**  For information about this choice, including consent withdrawal, please see our Privacy Policy

Reviewer #1: **Yes:**  Deeb Shrestha Dangol

Reviewer #2: No

**********

[NOTE: If reviewer comments were submitted as an attachment file, they will be attached to this email and accessible via the submission site. Please log into your account, locate the manuscript record, and check for the action link "View Attachments". If this link does not appear, there are no attachment files.]

To ensure your figures meet our technical requirements, please review our figure guidelines: https://journals.plos.org/plosone/s/figures

You may also use PLOS’s free figure tool, NAAS, to help you prepare publication quality figures: https://journals.plos.org/plosone/s/figures#loc-tools-for-figure-preparation.

NAAS will assess whether your figures meet our technical requirements by comparing each figure against our figure specifications.

Attachment Submitted filename: PONE-D-26-31923.pdf

# Author comment: Author response to Decision Letter 1

**DOI:** 10.1371/journal.pone.0357615.r002

30 Sep 2026

Response Letter to Reviewers

Manuscript PONE-D-26-31923

Women’s experiences of emergency post-abortion care at Kawempe National Referral Hospital, Uganda: a qualitative descriptive study

Dear Academic Editor and Reviewers,

We thank the Academic Editor and both reviewers for their careful and constructive engagement with our manuscript. We have revised the manuscript substantially in response to every point raised. Below we respond to each comment in turn, indicating the corresponding change in the manuscript. Line numbers refer to the clean revised manuscript submitted alongside this letter.

A. Journal Requirements

1. PLOS ONE style requirements, including file naming

Comment: Please ensure that your manuscript meets PLOS ONE’s style requirements, including those for file naming.

Response: We have reviewed the manuscript against the PLOS ONE Manuscript Body Formatting Guidelines and the Title/Author/Affiliations Formatting Guidelines. Heading levels, sentence-case headings, figure/table placement conventions, and reference formatting (numbered citation-sequence, Vancouver/ICMJE style) have been checked throughout. Files will be named per PLOS ONE convention at upload (Manuscript, Response to Reviewers, Revised Manuscript with Track Changes).

2. Data Availability Statement contact point

Comment: In the online submission form you indicate that your data is not available for proprietary reasons and have provided a contact point for accessing this data. Please note that your current contact point is a co-author on this manuscript. According to our Data Policy, the contact point must not be an author on the manuscript and must be an institutional contact, ideally not an individual. Please revise your data statement to a non-author institutional point of contact...

Response: The Data Availability Statement has been revised. The contact point is now Makerere University College of Health Sciences, Department of Obstetrics and Gynaecology, P.O. Box 7072, Kampala, Uganda (email: dean@chs.mak.ac.ug), which is an institutional contact and not a manuscript author. Requests remain subject to approval from SOMREC (reference Mak-SOMREC-2025-913). See Data availability statement, and the corresponding online submission form field, which has been updated to match.

3. Ethics statement location

Comment: Your ethics statement should only appear in the Methods section of your manuscript.

Response: Confirmed. The ethics statement appears only in Methods > Ethics statement. We have checked the Abstract, Results, Discussion, and Conclusions and confirmed no duplicate or partial ethics statement appears elsewhere.

4. Reviewer-suggested citations

Comment: If the reviewer comments include a recommendation to cite specific previously published works, please review and evaluate these publications to determine whether they are relevant and should be cited. There is no requirement to cite these works unless the editor has indicated otherwise.

Response: We reviewed both works suggested by Reviewer 2 and judged them relevant to strengthening the legal/bioethical framing of our Introduction. Both have been cited: Cioffi et al. (2023, Healthcare) and Ishola et al. (2021, Health Policy and Planning), now references 13 and 14. See Introduction, paragraph beginning “Uganda’s legal restriction of abortion is often described narrowly...”

B. Reviewer 1

Comment: Women’s experience in EPAC is very crucially related to the type of abortion undergone, possible complication undergone and the treatment options provided.

Response: We agree, and Table 1 has been expanded to report, for all sixteen participants: gestational age at presentation, presenting complication, treatment received, transfusion need, and referral status from another facility. Whether presentation followed miscarriage or induced abortion was not systematically captured in the clinical or interview records available for this analysis; this is now stated explicitly in Study population and data source, and its interpretive consequences (bounding stigma/guilt interpretations to narratives where this context was disclosed) are addressed in Strengths and limitations.

Comment: There are six broad areas mentioned as outline for the women’s experience, but the data mentioned does not reflect some of the areas (e.g., the referral system is mentioned of other health facilities where the women has encountered and not the experience of the referral from the study site).

Response: This is a fair characterisation of the data’s scope, and we have made that scope explicit rather than implicit. The interview guide (S1 File) contained no dedicated question on referral or transfer at any stage; pre-arrival referral content emerged through the open-ended opening question and its “step by step” probe, while referral or transfer between departments within KNRH once admitted (e.g., reception, the gynaecological emergency unit, and theatre) was neither asked about nor volunteered. We have revised the opening of Theme 5 (Structural barriers across the pathway of care) to state this directly, and added a corresponding point to Strengths and limitations noting this as a direction for future work — adding a dedicated question on intra-facility transfer specifically.

Comment: It also would have been good to see the PAC pathway experience by the level of providers as there are different cadres of providers providing the services in the study site.

Response: We agree this would strengthen the analysis, but provider cadre (e.g., consultant, resident, intern, midwife, nursing officer) was not systematically recorded during data collection; participants distinguish providers only descriptively (e.g., “the doctor”, “a female doctor”). We have added this as an explicit limitation in Strengths and limitations, stating plainly that this analysis cannot establish whether experiences of pain management, communication, or dignity differed systematically by provider cadre, as opposed to by individual provider or by moment in the care pathway, and flagging this as a design consideration for future EPAC research at KNRH.

C. Reviewer 2

We thank Reviewer 2 for an exceptionally thorough review. We address each point below in the order raised.

Comment: The designation of the study as phenomenological is not sufficiently substantiated, as the authors describe an inductive thematic analysis without specifying the phenomenological orientation or the analytical method actually employed.

Response: We agree and have removed the phenomenological designation throughout (title, abstract, Methods). The study is now described as a qualitative descriptive design using inductive thematic analysis, with an explicit statement that no specific phenomenological, grounded-theory, or narrative analytic tradition was adopted. See Study design.

Comment: The undifferentiated use of the term “post-abortion care”... The authors should therefore clarify whether the sample included women following miscarriage, induced abortion, or both; whether this information was collected; and how it was considered in the analysis.

Response: Clarified: this distinction was not systematically recorded in the clinical intake or interview protocol and was not used as an inclusion or exclusion criterion. We now state this directly in Study population and data source, and we have bounded our interpretation accordingly: stigma, guilt, and moral-vulnerability findings (Themes 2 and 4) are presented as grounded in the subset of narratives where participants themselves disclosed or implied the nature of the pregnancy loss, not as characterising the sample as a whole. This constraint is discussed further in Strengths and limitations.

Comment: The description of the Ugandan legal context should be expanded to consider not only the formal restrictiveness of abortion legislation but also its potential implications for the right to health, self-determination, and timely access to care... [suggested] https://doi.org/10.3390/healthcare11040520 and 10.1093/heapol/czab069.

Response: Expanded. A new paragraph in the Introduction (immediately following the existing legal/stigma paragraph) discusses restrictive abortion law as a determinant of health inequity and self-determination, citing Cioffi et al. (2023) for the bioethical/rights framing and Ishola et al. (2021) for empirical evidence that abortion law liberalisation is associated with improved health-service access and outcomes in LMICs, with restrictive regimes linked to delayed care-seeking and unsafe methods. Both are now references 13 and 14.

Comment: The statement that “approximately half of all abortions worldwide occur under unsafe conditions” should report the exact proportion provided by the cited source, the period to which the estimate refers, and the definition of “unsafe abortion” adopted... Rhetorical expressions such as “often the difference between life and death” should be replaced with more measured wording.

Response: Both corrected. The statistic now reads: “an estimated 45.1% of the 55.7 million abortions performed annually between 2010 and 2014 were unsafe, defined by WHO as a procedure to terminate a pregnancy carried out either by a person lacking the necessary skills or in an environment not meeting minimal medical standards, or both.” The “life and death” phrase has been replaced with: “timely access is strongly associated with reduced maternal morbidity and mortality.” See Introduction, paragraph 2.

Comment: The knowledge gap should be more clearly delineated... The existing studies should be briefly and critically synthesised, and the authors should specify which dimension remains insufficiently explored.

Response: A new paragraph has been added synthesising the existing Ugandan literature by what each strand actually covers (utilisation-focused quantitative studies vs. the one comparable qualitative satisfaction study) and stating explicitly what none of it addresses: EPAC specifically as a time-critical emergency presentation, and how women themselves weigh survival, dignity, and structural constraint against one another. See Introduction, paragraph immediately before “This study, therefore, explored...”.

Comment: The role of the Socio-Ecological Model (SEM) should also be described consistently... The authors should clarify whether the model informed the interview guide and coding process or was applied only after theme development.

Response: Clarified throughout. Study design and Data analysis now state explicitly that the SEM was applied post hoc, as an organising interpretive lens over inductively derived themes, not as an a priori framework shaping the interview guide or coding. The Introduction’s closing paragraph and the Discussion’s SEM subsection have been revised to match this framing consistently, and the Discussion now explicitly distinguishes conclusions grounded directly in participants' accounts from those introduced through the SEM’s organising structure.

Comment: If the authors intend to retain the phenomenological designation, they should specify the epistemological orientation... Otherwise, the study may be more accurately described as an exploratory qualitative study using thematic analysis. The EPAC population should also be clinically defined.

Response: As above, the study is now described as qualitative descriptive with inductive thematic analysis. The EPAC population is now clinically characterised in the expanded Table 1 (gestational age, presenting complication, treatment received, transfusion need, referral status). We chose qualitative description over the reviewer’s suggested alternative of "exploratory qualitative study" because "exploratory" describes a research purpose rather than a named analytic tradition, and does not specify how the data were analysed or what evidentiary standard the findings should be held to. Qualitative description, following Sandelowski (2000, 2010), is defined by staying close to the data with minimal researcher inference and producing a comprehensive, low-interpretation account in participants' own language, without committing to a specific theoretical lens; this matches both our analytic approach and the six fully-developed, sub-themed findings reported, which extend beyond a preliminary or scoping account.

Comment: The sampling strategy requires greater transparency... The authors should therefore explain how the final sample of 16 participants was reached and describe the operational criteria used to determine data saturation.

Response: Sampling strategy has been expanded: we now report that 24 women meeting eligibility criteria were approached, of whom 16 consented and 8 declined, and specify that maximal variation was operationalised using the characteristics summarised in Table 1. The saturation paragraph now states explicitly that saturation was assessed narratively (consistent with, but not applying, Guest, Namey and Chen’s quantitative tracking metrics), and that this is a qualitative judgement rather than a metrics-based determination.

Comment: Recruitment also appears to have occurred in connection with a scheduled review appointment, whereas some participants reported receiving no follow-up. This apparent inconsistency should be resolved.

Response: Resolved. Data collection now states explicitly that recruitment deliberately targeted the scheduled post-discharge review appointment (occurring around two weeks after discharge under standard clinical practice at the unit) rather than the point of discharge itself, and that this is consistent with the interval-to-interview data now reported. We have also added a limitation noting that participants interviewed at this point had already reached, or nearly reached, the standard follow-up window, which should be read alongside Theme 6’s account of perceived follow-up absence and the kind of follow up that the patient was looking for was perhaps a daily follow-up or after 2 days or at home and not necessarily a follow up review date which she saw as mandatory.

Comment: The manuscript also lacks an adequate account of researcher reflexivity. The authors should briefly describe the researchers' professional roles, their relationships with the institution and participants, and the strategies used to recognise and limit the influence of their assumptions.

Response: A new subsection, Researcher positionality, has been added to Methods. It describes all authors' clinical affiliation with KNRH/Makerere; that interviews were conducted by the corresponding author (a postgraduate trainee in the same department) with research assistants who were registered midwives employed at KNRH facilitating participant approach and consent; that this insider position extended to the interview encounter itself, not only to analysis; the single-analyst coding process and its senior-author review; and the reflexive journaling and bracketing procedure applied at three points (a pre-data-collection session recording assumptions and clinical background, individual post-interview reflexive journals kept separately by each interviewer, and analytic memos reviewed in team coding meetings), used specifically to check clinically-derived codes against participants' own words.

Comment: Table 1 should also report... the principal clinical characteristics: miscarriage or induced abortion, where known; gestational age; presenting complication; treatment received; need for transfusion; referral from another facility; and interval between discharge and interview.

Response: Table 1 has been expanded with all of these variables except miscarriage/induced abortion status, which was not captured (explained above and in Study population and data source). The added rows report, for all sixteen participants: gestational age at presentation, presenting complication, treatment received, transfusion need, referral status from another facility, and interval between discharge and interview.

Comment: Statements regarding costs also require clarification... The quotation referring to money required in connection with a blood transfusion does not appear fully consistent with the description of EPAC as free of charge.

Response: Clarified. A new passage in Theme 5 distinguishes formal facility charges (ultrasound scans, medications out of stock at the pharmacy, consistent with EPAC itself being officially free) from informal payment: in the account concerning blood transfusion, money was given directly to health workers not as a billed fee for the blood product or its screening, but as an informal incentive intended to motivate staff to act quickly in sourcing blood for an urgent transfusion. We note this is consistent with informal payment practices documented elsewhere in under-resourced public health systems.

Comment: Particular attention should also be paid to the participant reporting symptoms potentially suggestive of infection. The authors should clarify whether the research protocol included clinical assessment or referral procedures for health concerns identified during an interview.

Response: Clarified in Theme 6: the study protocol included a defined procedure for referring participants for clinical assessment where concerning symptoms were disclosed mid-interview; no case during data collection required this procedure to be invoked.

Comment: The Discussion offers an interesting interpretation... but several conclusions extend beyond what the data can support... Expressions such as “fear-driven reordering”, “coercive”, “produced”, and “the SEM resolved” should therefore be replaced with more cautious wording.

Response: All four phrases have been revised. “Fear-driven reordering” is replaced with a direct description of acute mortal threat leading women to prioritise survival, temporarily subordinating relational and procedural quality dimensions. The passage previously implying coercion now describes judgmental communication as “compounded disrespect” given that women in acute emergency presentations cannot exit the care relationship. “The SEM resolved” is replaced with “The SEM provided a coherent multi-level account of the conditions under which clinical success and relational failure simultaneously occurred.” We reviewed the Discussion throughout for similarly unsupported causal language.

Comment: The comparison with the literature should clarify which findings are genuinely novel rather than stating generically that the study confirms and extends previous evidence.

Response: The Discussion’s engagement with prior literature (Baum, Ouedraogo, Cleeve, Lythgoe, Udho, Stillman, Kabunga, and others) has been reviewed to ensure each comparison states specifically what this study confirms, extends, or adds, rather than a generic confirms-and-extends framing; see Discussion, paragraphs 2–6.

Comment: The Limitations section should more fully acknowledge potential selection and social desirability biases, the influence of the setting and researchers, translation-related issues, the clinical heterogeneity of the sample, and the absence of corroborating clinical or organisational data.

Response: Strengths and limitations has been substantially expanded to address: the two-stage recruitment process potentially under-sampling women most fearful of re-engaging with the health system; the 33% refusal rate (8 of 24) as an uncharacterised source of possible selection bias; institutional social-desirability pressure arising from interviewing on hospital premises during a dependent clinical relationship; a power differential between the research team and a participant group of whom 62.5% had at most lower secondary education; the timing confound between recruitment window and standard follow-up scheduling; unrecorded geographic origin and religious affiliation; unrecorded uterine evacuation procedure type; the miscarriage/induced abortion distinction; translation conducted by the interviewers themselves rather than independent translators; and the intra-facility referral and provider-cadre gaps raised by Reviewer 1.

Comment: The Conclusions adequately reflect the central message of the study but should be explicitly confined to the experiences of the 16 participants. The assertion that post-discharge recovery was largely unstructured should be tempered, while stigma should not be characterised as a “direct determinant” in the absence of causal evidence.

Response: Conclusions have been revised: the opening sentence now reads “Among the sixteen women interviewed...” and explicitly scopes the survival-is-necessary-but-not-sufficient claim to this sample. The recovery statement now reads “recovery experienced by these participants as largely unstructured” rather than a general claim. “Stigma as a direct determinant of care quality” has been replaced with language noting that stigma shaped how care was communicated and experienced, while its causal contribution to objective care quality was not directly tested.

Reference cited in this letter

Sandelowski, M., 2000. Whatever happened to qualitative description? Research in Nursing & Health, 23(4), pp.334–340.

Sandelowski, M., 2010. What’s in a name? Qualitative description revisited. Research in Nursing & Health, 33(1), pp.77–84.

Closing

We believe these revisions have substantially strengthened the manuscript’s methodological transparency, the precision of its claims, and its engagement with the existing literature and legal context. We thank the Academic Editor and both reviewers again for their time and constructive guidance, and hope the revised manuscript now meets PLOS ONE’s criteria for publication.

On behalf of all authors,

Kawungu Saad Sessimba (corresponding author)

Attachment Submitted filename: Response to Reviewers.docx

# Peer review: Decision Letter 1

**DOI:** 10.1371/journal.pone.0357615.r003

**Authors:** Andrea Cioffi

12 Oct 2026

Dear Dr. Saad Sessimba,

Thank you for submitting your manuscript to PLOS One. After careful consideration, we feel that it has merit but does not fully meet PLOS One’s publication criteria as it currently stands. Therefore, we invite you to submit a revised version of the manuscript that addresses the points raised during the review process.

Please, see reviewer’s comments below.

Please submit your revised manuscript by Nov 11 2026 11:59PM. If you will need more time than this to complete your revisions, please reply to this message or contact the journal office at plosone@plos.org. When you're ready to submit your revision, log on to https://www.editorialmanager.com/pone/ and select the 'Submissions Needing Revision' folder to locate your manuscript file.

- A letter that responds to each point raised by the academic editor and reviewer(s). You should upload this letter as a separate file labeled 'Response to Reviewers'.
- A marked-up copy of your manuscript that highlights changes made to the original version. You should upload this as a separate file labeled 'Revised Manuscript with Track Changes'.
- An unmarked version of your revised paper without tracked changes. You should upload this as a separate file labeled 'Manuscript'.

If you would like to make changes to your financial disclosure, please include your updated statement in your cover letter. Guidelines for resubmitting your figure files are available below the reviewer comments at the end of this letter.

If applicable, we recommend that you deposit your laboratory protocols in protocols.io to enhance the reproducibility of your results. Protocols.io assigns your protocol its own identifier (DOI) so that it can be cited independently in the future. For instructions see: https://journals.plos.org/plosone/s/submission-guidelines#loc-laboratory-protocols. Additionally, PLOS One offers an option for publishing peer-reviewed Lab Protocol articles, which describe protocols hosted on protocols.io. Read more information on sharing protocols at https://plos.org/protocols?utm_medium=editorial-email&utm_source=authorletters&utm_campaign=protocols.

As the corresponding author, your ORCID iD is verified in the submission system and will appear in the published article. PLOS supports the use of ORCID, and we encourage all coauthors to register for an ORCID iD and use it as well. Please encourage your coauthors to verify their ORCID iD within the submission system before final acceptance, as unverified ORCID iDs will not appear in the published article. *Only*  the individual author can complete the verification step; PLOS staff *cannot*  verify ORCID iDs on behalf of authors.

We look forward to receiving your revised manuscript.

Kind regards,

Andrea Cioffi

Academic Editor

PLOS One

**Journal Requirements:**

1. If the reviewer comments include a recommendation to cite specific previously published works, please review and evaluate these publications to determine whether they are relevant and should be cited. There is no requirement to cite these works unless the editor has indicated otherwise.

2. Please review your reference list to ensure that it is complete and correct. If you have cited papers that have been retracted, please include the rationale for doing so in the manuscript text, or remove these references and replace them with relevant current references. Any changes to the reference list should be mentioned in the rebuttal letter that accompanies your revised manuscript. If you need to cite a retracted article, indicate the article’s retracted status in the References list and also include a citation and full reference for the retraction notice.

[Note: HTML markup is below. Please do not edit.]

Reviewers' comments:

Reviewer’s Responses to Questions

**Comments to the Author**

Reviewer #2: (No Response)

**********

2. Is the manuscript technically sound, and do the data support the conclusions?

Reviewer #2: Yes

**********

3. Has the statistical analysis been performed appropriately and rigorously?

Reviewer #2: I Don't Know

**********

4. Have the authors made all data underlying the findings in their manuscript fully available?

The PLOS Data policy

Reviewer #2: Yes

**********

5. Is the manuscript presented in an intelligible fashion and written in standard English?

Reviewer #2: Yes

**********

Reviewer #2: I would first like to thank the Editor for the opportunity to review this revised manuscript.

I also thank the Authors for their detailed responses to my previous comments and for the considerable effort they have put into revising their work.

The manuscript has improved substantially, particularly in terms of methodological clarity, transparency of the analytical process, and interpretation of the findings. Most of my previous concerns have been satisfactorily addressed. Having reviewed the revised manuscript, however, I believe a few points still need clarification.

(All line numbers below refer to the clean version of the revised manuscript)

First, the Authors state in their response letter that Table 1 has been expanded to include the interval between discharge and interview, but this information does not appear in the revised table. I also noticed an inconsistency concerning the treatment procedures. Table 1 specifies the treatment received by all sixteen participants (manual vacuum aspiration, misoprostol-only management, and sharp curettage), whereas the Strengths and limitations section (lines 642–644) states that the type of uterine evacuation procedure was not systematically recorded. I would ask the authors to clarify this discrepancy.

Regarding the costs reported by participants, Theme 5 describes the money associated with a blood transfusion as an informal payment made directly to healthcare workers to expedite care. However, the participant’s account quoted in the manuscript does not seem to support this specific interpretation. Unless further details from the interview substantiate this claim, I would suggest revising the wording to distinguish more clearly between what the participant actually reported and the authors' interpretation.

I would also appreciate some clarification regarding participant IDI_6, who reported persistent lower abdominal pain and what she described as potentially purulent discharge during the interview (lines 471–478). The authors explain that a referral procedure was available for participants reporting concerning symptoms, but state that no case required its activation. Although these symptoms do not necessarily indicate an infection, it remains unclear why they were not considered sufficient to warrant further clinical assessment.

Finally, the description of data saturation remains somewhat unclear. Since the manuscript now identifies the study as a secondary thematic analysis of interviews collected within a broader study, it would be useful to specify whether saturation informed the original recruitment process or was assessed retrospectively. I would also suggest distinguishing more clearly between the absence of proactive contact after discharge and the absence of scheduled follow-up appointments, as these are not necessarily equivalent and may have different implications for the interpretation of the findings.

Overall, I appreciate the substantial improvements made to the manuscript and consider the authors' responses to most of my previous comments satisfactory. The remaining issues are relatively limited and, in my view, can be addressed without major changes to the study. Once these points have been clarified, I believe the manuscript could be considered suitable for publication.

**********

what does this mean?). If published, this will include your full peer review and any attached files.

If you choose “no”, your identity will remain anonymous but your review may still be made public.

**Do you want your identity to be public for this peer review?**  For information about this choice, including consent withdrawal, please see our Privacy Policy

Reviewer #2: No

**********

[NOTE: If reviewer comments were submitted as an attachment file, they will be attached to this email and accessible via the submission site. Please log into your account, locate the manuscript record, and check for the action link "View Attachments". If this link does not appear, there are no attachment files.]

To ensure your figures meet our technical requirements, please review our figure guidelines: https://journals.plos.org/plosone/s/figures

You may also use PLOS’s free figure tool, NAAS, to help you prepare publication quality figures: https://journals.plos.org/plosone/s/figures#loc-tools-for-figure-preparation.

NAAS will assess whether your figures meet our technical requirements by comparing each figure against our figure specifications.

# Author comment: Author response to Decision Letter 2

**DOI:** 10.1371/journal.pone.0357615.r004

15 Oct 2026

Response to the Academic Editor and Reviewer 2

We thank the Academic Editor and Reviewer 2 for their careful second-round comments. A fuller point-by-point response is uploaded as “Response to Reviewers”. A summary follows. Line numbers refer to the clean Manuscript file.

Journal Requirements

Reviewer-suggested citations. Reviewers did not recommend specific publications in this round, so we added no citations on that basis.

Reference list. We checked all 37 references for completeness and accuracy. No reference was added, removed or replaced. We also screened all 37 against the Retraction Watch database and found no retracted articles.

Reviewer 2

Statistical analysis. This is a qualitative study and no statistical tests were run. Table 1 reports participant counts only.

Table 1: interval between discharge and interview. Our first response listed the interval as a row added to Table 1, but the row was not in the submitted table. We removed it because the interval is the same for every participant, and we should have updated the letter. Every participant was interviewed at least 14 days after discharge, and the exact number of days beyond 14 was not recorded. We therefore left Table 1 unchanged and state this in the text: the criterion is in Sampling strategy (lines 214–216), and Data collection (lines 246–247) now says all sixteen participants were interviewed at least 14 days after discharge.

Treatment received versus the limitations section. The two statements contradicted each other. Table 1 reports the procedure for all 16 participants, so we deleted the sentence saying it was not systematically recorded. The remaining limitation (lines 655–659) is narrower: procedure type was not used as an analytic variable, and with 16 participants the Theme 2 pain findings cannot be compared across procedures.

Theme 5: money paid in connection with a blood transfusion. We agree that the quotation alone does not show where the money went. The explanation now sits directly under the IDI_2 quotation in Theme 3 (lines 390–397). It states that, under hospital policy, EPAC at KNRH is free of charge except for radiological services, which all patients pay for out of pocket. It says IDI_2 did not state who received the money or what it was for. It then adds a second statement from the same interview, set out as a quotation in her words. Read together, these lead us to interpret the money as an attempt to speed up care, not as payment for blood or tests. Study setting (lines 189–191) now states the policy. The Theme 5 paragraph (lines 445–449) is now a short pointer to Theme 3.

IDI_6. Her full quotation is restored (lines 470–474). Theme 6 (lines 475–482) now states that she was interviewed at her scheduled review appointment at the gynaecological emergency unit, and that the protocol provided for referral of any participant who disclosed concerning symptoms. It also states that she agreed to continue the interview because her symptoms were not too severe for her, and that, with her consent, the reviewing clinician was made aware. We deleted the earlier statement that no case required the referral procedure, in Theme 6 and in Strengths and limitations (lines 662–664).

Data saturation. Saturation informed recruitment. Interviews were transcribed and coded while fieldwork was still under way, and fieldwork continued until new interviews produced the same information as earlier ones, after which no further interviews were conducted. Sampling strategy now says this (lines 227–238), and that the judgement is narrative and did not use quantitative metrics.

Absence of proactive contact versus absence of scheduled follow-up. We made this distinction in four places: Theme 6 (lines 467–469 and 475–490), Table 2 (page 17), the Discussion (line 570), and Strengths and limitations (lines 635–637). Participants described no contact from the hospital after discharge. Every participant was interviewed at a scheduled review appointment, so the data speak to missing proactive contact and not to missing appointments.

Supporting file. S2 File (COREQ) has been updated so its page numbers match the revised clean manuscript, and its responses on saturation, coding, field notes and sample characteristics match the revised text.

Attachment Submitted filename: Response_to_Reviewers_auresp_2.docx

# Peer review: Decision Letter 2

**DOI:** 10.1371/journal.pone.0357615.r005

**Authors:** Andrea Cioffi

22 Oct 2026

Women’s experiences of emergency post-abortion care at Kawempe National Referral Hospital, Uganda: a qualitative descriptive study

PONE-D-26-31923R2

Dear Dr. Saad Sessimba,

We’re pleased to inform you that your manuscript has been judged scientifically suitable for publication and will be formally accepted for publication once it meets all outstanding technical requirements.

Within one week, you’ll receive an e-mail detailing the required amendments. When these have been addressed, you’ll receive a formal acceptance letter and your manuscript will be scheduled for publication.

An invoice will be generated when your article is formally accepted. Please note, if your institution has a publishing partnership with PLOS and your article meets the relevant criteria, all or part of your publication costs will be covered. Please make sure your user information is up-to-date by logging into Editorial Manager at Editorial Manager® and clicking the ‘Update My Information' link at the top of the page. For questions related to billing, please contact billing support.

If your institution or institutions have a press office, please notify them about your upcoming paper to help maximize its impact. If they’ll be preparing press materials, please inform our press team as soon as possible -- no later than 48 hours after receiving the formal acceptance. Your manuscript will remain under strict press embargo until 2 pm Eastern Time on the date of publication. For more information, please contact onepress@plos.org.

Kind regards,

Andrea Cioffi

Academic Editor

PLOS One

Additional Editor Comments (optional):

Reviewers' comments:

Reviewer’s Responses to Questions

**Comments to the Author**

Reviewer #2: All comments have been addressed

**********

2. Is the manuscript technically sound, and do the data support the conclusions?

Reviewer #2: Yes

**********

3. Has the statistical analysis been performed appropriately and rigorously?

Reviewer #2: N/A

**********

4. Have the authors made all data underlying the findings in their manuscript fully available?

The PLOS Data policy

Reviewer #2: Yes

**********

5. Is the manuscript presented in an intelligible fashion and written in standard English?

Reviewer #2: Yes

**********

Reviewer #2: First of all, I would like to thank the Editor for the opportunity to review this manuscript once again.

I would also like to thank the Authors for their detailed responses and for the attention they have given to the comments raised during the previous rounds of review.

I believe that this latest revision has satisfactorily addressed the concerns that remained in the previous version. In particular, the authors have clarified methodological aspects relating to sampling and data saturation, resolved inconsistencies in the description of treatment procedures, and provided more precise explanations of participant accounts that had raised clinical and interpretative questions. I also appreciate the clearer distinction between what was directly reported by participants and the authors' subsequent interpretations, as well as the more careful consideration of the limitations of the available data.

Overall, the manuscript now shows greater internal consistency and methodological transparency. The authors have addressed the main concerns raised in my previous reviews and made substantive changes that have strengthened the scientific quality of the work.

Although the study remains subject to the limitations of its qualitative design and sample, I believe these are now adequately acknowledged and do not undermine its overall value.

In light of the improvements made, I consider the manuscript suitable for publication and recommend its acceptance.

**********

what does this mean?). If published, this will include your full peer review and any attached files.

If you choose “no”, your identity will remain anonymous but your review may still be made public.

**Do you want your identity to be public for this peer review?**  For information about this choice, including consent withdrawal, please see our Privacy Policy

Reviewer #2: No

**********

# Editor report: Acceptance letter

**DOI:** 10.1371/journal.pone.0357615.r006

**Authors:** Andrea Cioffi

PONE-D-26-31923R2

PLOS One

Dear Dr. Saad Sessimba,

I'm pleased to inform you that your manuscript has been deemed suitable for publication in PLOS One. Congratulations! Your manuscript is now being handed over to our production team.

At this stage, our production department will prepare your paper for publication. This includes ensuring the following:

* All references, tables, and figures are properly cited

* All relevant supporting information is included in the manuscript submission,

* There are no issues that prevent the paper from being properly typeset

You will receive further instructions from the production team, including instructions on how to review your proof when it is ready. Please keep in mind that we are working through a large volume of accepted articles, so please give us a few days to review your paper and let you know the next and final steps.

Lastly, if your institution or institutions have a press office, please let them know about your upcoming paper now to help maximize its impact. If they'll be preparing press materials, please inform our press team within the next 48 hours. Your manuscript will remain under strict press embargo until 2 pm Eastern Time on the date of publication. For more information, please contact onepress@plos.org.

You will receive an invoice from PLOS for your publication fee after your manuscript has reached the completed accept phase. If you receive an email requesting payment before acceptance or for any other service, this may be a phishing scheme. Learn how to identify phishing emails and protect your accounts at https://explore.plos.org/phishing.

If we can help with anything else, please email us at customercare@plos.org.

Thank you for submitting your work to PLOS One and supporting open access.

Kind regards,

PLOS One Editorial Office Staff

on behalf of

Dr. Andrea Cioffi

Academic Editor

PLOS One
